# Efficacy and safety of GLP-1 agonists in Parkinson’s disease: a systematic review and meta-analysis of randomized controlled trials

**DOI:** 10.1007/s00210-025-03932-3

**Published:** 2025-03-11

**Authors:** Mark Messak, Ahmed Abdelmageed, Abdelrahman A. Senbel, Youssef A. Khattab, Youssef Mandour, Omar Shaker, Ahmed Hamed Rehan, Samir Oransa, Mohamed Nasr, Abdullah Emad Shabeeb, Ziyad Rezq, Fares Hossam, Moaz Elsayed Abouelmagd

**Affiliations:** 1 Faculty of Medicine, Helwan University, Helwan, Egypt; 2 Faculty of Medicine, Mansoura University, Mansoura, Egypt; 3 Faculty of Medicine, Cairo University, Cairo, Egypt; 4 Faculty of Medicine, Delta University, Gamasa, Egypt; 5 Medical Research Group of Egypt, Negida Academy LLC, Arlington, MA USA

**Keywords:** Exenatide, Glucagon-like peptide-1, Lixisenatide, Parkinson’s disease

## Abstract

Recent trends suggest exploring the repurposing of different drugs for Parkinson’s disease patients (PD). One of these drugs is Glucagon-like peptide-1 (GLP-1), a medication used to treat type 2 diabetes mellitus. This systematic review and meta-analysis aimed to evaluate the efficacy and safety of different forms of GLP-1 agonists on motor and non-motor functions of PD patients during ON-medication and OFF-medication states. A literature search was conducted through PubMed, Scopus, Web of Science, OVID, Cochrane Central, and Google Scholar using relevant keywords. Quality assessment was performed using the Risk of Bias-2 (RoB-2) domains. Statistical analysis included calculating the mean difference (MD) and the corresponding 95% confidence intervals (CIs) using Review Manager 5.4.1. Four randomized controlled trials (RCTs) testing three different forms of GLP-1 agonists with a total of 514 patients were included in the study. GLP-1 agonists significantly improved motor function during the OFF-medication state (MD =  − 3.29, 95% CI [− 5.17 to − 1.42], *P* = 0.0006). It does not show improvement in quality of life assessed by PDQ-39 (MD =  − 0.54, 95% CI [− 2.07 to 0.99], *P* = 0.49). None of the adverse effects stated in the RCTs were higher in the GLP-1 agonists group except for nausea (RR = 1.98, *P* = 0.0008), vomiting (RR = 6.65, *P* = 0.0008), constipation (RR = 1.45, *P* = 0.01), and weight loss (RR = 2.11, *P* = 0.03). This systematic review and meta-analysis provide evidence that GLP-1 agonists could improve the motor function of PD patients. However, safety is still of concern. Further high-quality studies with standardized protocols and larger sample sizes are needed to confirm our findings.

**Supplementary Information:**

The online version contains supplementary material available at 10.1007/s00210-025-03932-3.

## Introduction

Parkinson’s disease (PD) is the second most common progressive, age-related movement disorder worldwide (Mhyre et al. 2012; Zafar and Yaddanapudi 2023). It is estimated that by 2040, approximately 17 million people will be affected by PD (Dorsey et al. 2018). PD is characterized by motor and non-motor symptoms. Motor symptoms are the most important clinical features of PD; they are manifested by resting tremors, rigidity, and bradykinesia (Chang et al. 1995; Fritsch et al. 2012; Moustafa et al. 2016). Non-motor symptoms may appear in the earliest stages as weight loss, constipation, depression, anosmia, sleep problems, and orthostatic hypotension (Kim and Sung 2015; Löhle et al. 2009). The main cause of the disease is degeneration in the substantia nigra pars compacta neurons which are nerve cells responsible for producing dopamine (Dauer and Przedborski 2003).

Glucagon-like peptide-1 (GLP-1) is an incretin hormone secreted from the intestine as a result of meal ingestion (Vilsbøll et al. 2012). GLP-1 agonists are FDA-approved drugs used in the treatment of type 2 diabetes mellitus and obesity as they increase insulin release, decrease glucagon production, enhance proliferation of pancreatic beta cells, and delay gastric emptying (Collins and Costello 2024). GLP-1 agonists were found to have neuroprotective features because of the following: (1) They reduce the levels of amyloid precursor protein and amyloid-beta peptide, (2) GLP-1 agonists can enhance nerve growth factor-initiated differentiation, (3) They reduce ibotenic acid-induced exhaustion of choline acetyltransferase immunoreactivity in cholinergic neurons, (4) They modulate calcium responses to glutamate and membrane depolarization. These features nominated GLP-1 agonists as a promising candidate for the treatment of neurodegenerative diseases including PD (Gilman et al. 2003; Perry et al. 2003; Perry et al. 2002a, b; Perry et al. 2002a, b).

Results of preclinical studies using GLP-1 agonists for PD showed that they have a significant role in improving PD symptoms. In rats, they had anti-inflammatory neuronal effects, increased striatal dopamine, and decreased malondialdehyde and tumor necrosis factor alpha amount (Aksoy et al. 2017; Elbassuoni and Ahmed 2019). In another study on a mouse model, they had the ability to improve motor function and protect neurons against degeneration (Li et al. 2009).

Athauda et al. conducted a randomized controlled trial (RCT) testing exenatide, a GLP-1 agonist, on moderate PD patients by subcutaneous injection once weekly for 48 weeks, its findings showed improvement in the exenatide group over the placebo group in the motor function assessed by MDS-UPDRS III at OFF-medication state with adjusted mean difference of − 3.5 points (Athauda et al. 2017). Meissner et al. study, another RCT that experimented with subcutaneous Lixisenatide once a day for 12 months, found significant improvement in symptoms between Lixisenatide and placebo groups (Meissner et al. 2024). A previous systematic review conducted by Cochrane Collaboration reported low-certainty evidence that using exenatide improves the motor functions of PD patients (Mulvaney et al. 2020). More studies have been published since then with conflicting results regarding the progression of the motor disability of PD patients (McGarry et al. 2024; Meissner et al. 2024) and therefore an update systematic review and meta-analysis is needed.

This systematic review and meta-analysis aim to fill the gap in understanding the true effect of GLP-1 agonists on motor function in PD patients. In addition, we aim to evaluate the impact of these drugs on their mood and quality of life.

## Methods

We followed the PRISMA guidelines for Systematic reviews and meta-analysis while reporting this manuscript (Page et al. 2021). We were adherent to the Cochrane Handbook of Systematic Reviews of Interventions version 6 while conducting this study (Higgins et al. 2019a, b, c, d). This study was prospectively registered on PROSPERO with the register number CRD42024619237.

### Eligibility criteria

Studies satisfying the following inclusion criteria were included in the study: (1) RCTs comparing GLP-1 agonists with placebo or conventional treatment, (2) studies whose population is PD patients of any stage, (3) studies measuring at least one of the following outcomes: MDS-UPDRS Parts I – IV, MADRS, NMSS, LED, and PDQ-39. We excluded studies not written in English language, studies on animal models, study designs not a randomized controlled trial, conference abstracts, protocols, commentary articles, post-hoc analysis articles, and papers reporting other neurodegenerative diseases other than PD.

### Literature search strategy

We conducted a computer literature search of PubMed, Scopus, Web of Science, OVID, Cochrane Central, and Google Scholar. We used the following query in our search: ((Glucagon-like peptide-1) OR (GLP-1)) AND (Parkinson’s disease) AND ((Randomized Controlled Trial) OR (RCT)) and for sensitive search strategy we used MeSH database in the following query: (“Glucagon-Like Peptide-1 Receptor Agonists”[Mesh]) AND “Parkinson Disease”[Mesh]). The full search strategy we used in different databases is found in Table 1 in the supplementary materials.

### Selection process and data extraction

Eligibility screening was performed in two steps using Rayyan software (Ouzzani et al. 2016). The first step was the title and abstract screening of all retrieved papers. The second step was full-text screening for those that passed the first step of screening to assess the eligibility for meta-analysis. Five authors were responsible for this selection process.

Three authors extracted the data independently using an online extraction form. The extracted data includes the following: (1) characters of the study design, (2) characters of the study population, (3) risk of bias-2 domains, and (4) study outcomes: Mean change from baseline of the following: MDS-UPDRS I, MDS-UPDRS II, MDS-UPDRS III ON-medication, MDS-UPDRS III OFF-medication, MDS-UPDRS IV, MADRS, NMSS, LED, and PDQ-39.

### Risk of bias assessment

Eight authors evaluated the quality of the included studies independently according to the Cochrane Handbook of Systematic Reviews of Interventions version 6 (Higgins et al. 2019d). We used the Risk of Bias assessment tool-2 (RoB-2) mentioned in the same book chapter 8. The RoB-2 tool comprises five bias domains: (1) bias arising from the randomization process, (2) bias due to deviations from intended interventions, (3) bias due to missing outcome data, (4) bias in measurement of the outcome, (5) bias in selection of the reported results. For visualizing our risk of bias assessment, we used the robvis web app (McGuinness and Higgins 2021).

### Measures of treatment effect of GLP-1 agonists and data synthesis

The four MDS-UPDRS subscales are the primary outcomes of our study. The MDS-UPDRS is composed of four parts: I (non-motor experiences of daily living), II (motor experiences of daily living), III (motor function), and IV (motor complications). It is a semi-objective scale as it depends on both clinician-based assessment and patient-reporting symptoms (Goetz et al. 2008). Our secondary outcomes were PDQ-39 for assessing quality of life, NMSS for assessing non-motor symptoms, MADRS for assessing depression, and L-dopa equivalent dose (Chaudhuri et al. 2007; Jenkinson et al. 1997; Montgomery and Asberg 1979). These scales, except LED, are subjective scales that depend on the patient to report the symptoms. Changes from baseline in MDS-UPDRS sub-scores, LED, MADRS, NMSS, and PDQ-39 were pooled as mean difference (MD) in a meta-analysis model. We used risk ratio to assess the effect estimate of the adverse events. To prevent unit-of-analysis error, we combined the two interventional groups of McGarry et al. (2024) to create a single pair-wise comparison as recommended by the Cochrane Handbook of Systematic Reviews of Interventions version 6 (Part 4, Chapter 23.3.4) (Higgins et al. 2019a) using the formula stated in part 2, chapter 6.5.2.10 of the same book (Higgins et al. 2019b). We used the calculator of Review Manager Version 5.4.1 for Windows. For adverse events, we combined the two interventional groups.

When the standard deviation of change in one of the outcomes was not provided, we calculated it from a 95% confidence interval or by imputing standard deviations for changes from baseline using correlation coefficient (r) = 0.85 suggested by Athauda et al. study (2017) or by calculating it from the standard error (SE). We used meta-analysis accelerator for performing the calculations (Abbas et al. 2024).

$$\begin{array}{c}SD=SE\times \sqrt{N}\\ SD=\sqrt{N}\times \left(Upper\, limit-lower\, limit\right)/ 3.92\\ \begin{array}{c}{Mean}_{Change}={Mean}_{Final}-{Mean}_{Baseline}\\ {SD}_{Change}=\sqrt{{SD}_{Baseline}^{2}+{SD}_{Final}^{2}-\left(2\times r\times {SD}_{Baseline}\times {SD}_{Final}\right)}\end{array}\end{array}$$

### Heterogeneity assessment

Heterogeneity was assessed by visual inspection of the *I*^2^ and Chi^2^ tests found in the forest plots. In case of significant heterogeneity (*P*-value < 0.1), sensitivity analysis was performed by exclusion of each study independently.

### Publication bias

According to Egger and his colleague’s paper (Egger et al. 1997), publication bias needs at least 10 papers in order to be performed. Therefore, in our study, we could not assess the publication bias using Egger’s funnel plot asymmetry.

## Results

### Search results

Our search retrieved 691 unique studies, 184 were duplicated studies and after resolving, 586 studies were subjected to title and abstract screening. Following the title and abstract screening, 556 studies were excluded and only 30 were retrieved and screened for eligibility.

**Fig. 1:**
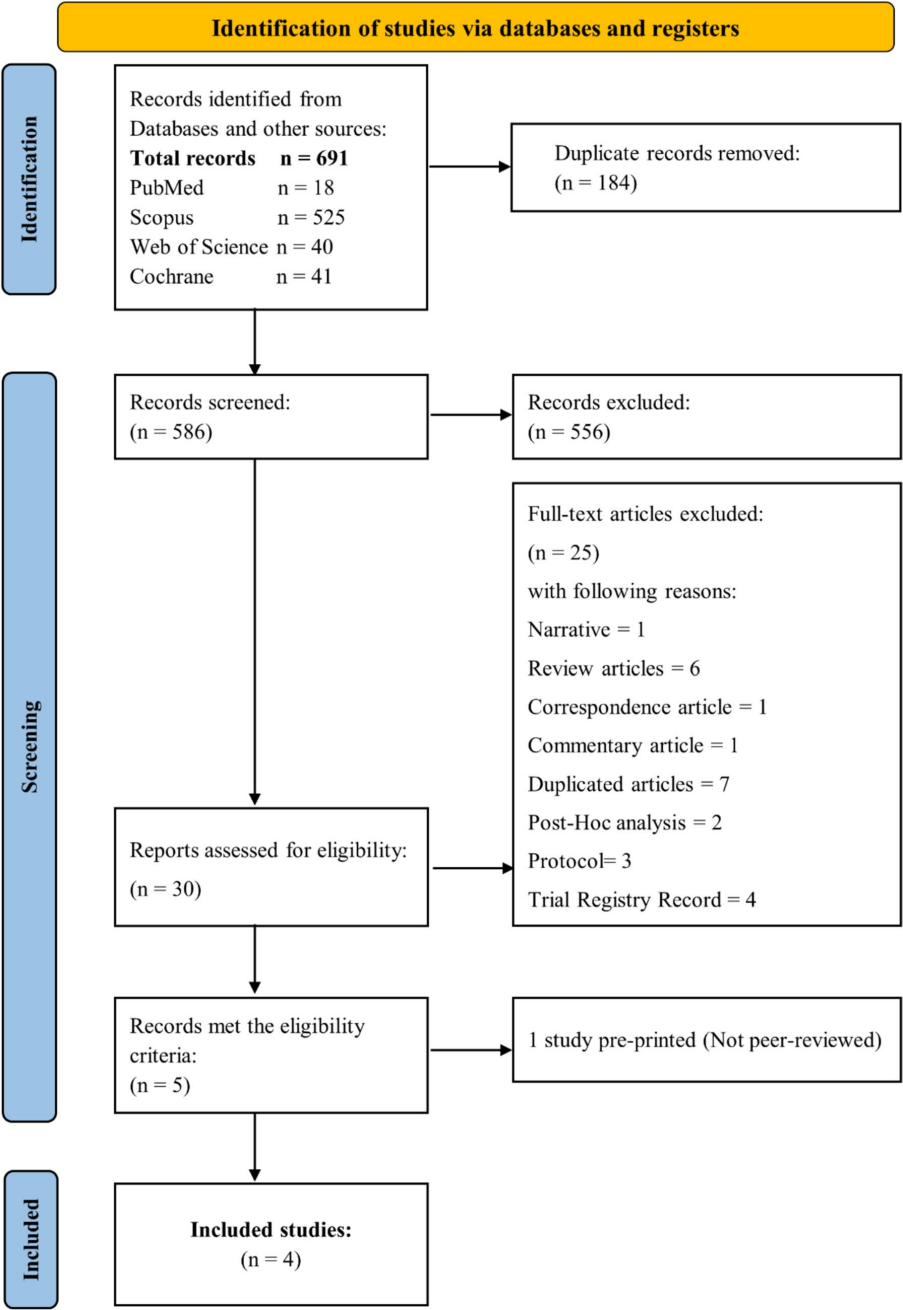
PRISMA flow diagram of studies selection process

Of the 30 full-text studies, 25 studies were excluded, one paper was found to be pre-printed (not peer-reviewed) (Hogg et al. 2022) and four RCTs (*n* = 514 patients) were included in this study. The reasons for excluding the studies were stated in the PRISMA flow diagram. The pre-printed study was subjected to meta-analysis; however, our results, discussion, and conclusion in this paper were conducted according to the four included and peer-reviewed RCTs. Figure 1 shows the PRISMA flow diagram for the identification and selection of the studies.

Of the included studies, two of them were conducted in the UK (Athauda et al. 2017; Aviles-Olmos et al. 2013), one in the USA (McGarry et al. 2024), and one in France (Meissner et al. 2024) summary of their designs and main results is shown in Table 1 and the baseline characteristics of their populations are shown in Table 2.

**Table 1:** Summary of the included studies

Study ID	Study design	Dose & route of administration	Evaluation time points	Population	Intervention	Comparator	Key findings
Athauda et al.; 2017	Randomized, double-blind, placebo-controlled, parallel-group, single-center trial	Subcutaneously, 2 mg	Baseline, 3, 6, 9, 12, and 15 months	62 patients with idiopathic PD as measured by Queen Square Brain Bank criteria	Exenatide	Placebo	Exenatide had improved UPDRS part 3 off-medication by 1.0 points (95% CI –2.6 to 0.7). However, the long-lasting effect of the drug is uncertain
Aviles-Olmos et al.; 2013	Phase 2, randomized, single-blind controlled trial design	Subcutaneously, 5 µg for 1 month then 10 µg for the rest of 12 months	Baseline, 6, 12, and 14 months	45 moderate PD patients	Exenatide	Conventional treatment	Compared to the control group, the drug showed clinically relevant improvements in PD patients across motor and cognitive measures. However, weight loss is a common side effect
McGarry et al.; 2024	Randomized, double-blind, placebo-controlled study	Subcutaneously, either 2.5 mg or 5.0 mg of NLY01	Baseline and 9 months	255 early untreated PD patients	NLY01	Placebo	Compared to placebo, NLY01 did not show any significant improvement in motor and non-motor symptoms of PD patients. Further studies are needed
Meissner et al.; 2024	Investigator-initiated, phase 2, multicenter, double-blind, parallel-group, randomized, placebo-controlled trial	Subcutaneously, an initial dose of 10 µg per day for 14 days, then 20 µg per day to the end of study	Baseline, 6, 12, and 14 months	156 PD patients diagnosed in less than 3 years	Lixisenatide	Placebo	Compared to placebo, Lixisenatide showed positive results in motor disability at 12 months, but it was associated with some GI disorders. Longer trials with larger sample sizes are recommended to investigate its efficacy in PD treatment

**Table 2:** Baseline characters of the included studies. *SD* standard deviation

Study ID	Group	Sample size	Age Mean (SD)	Gender Male (%)	MDS-UPDRS I Mean (SD)	MDS-UPDRS II Mean (SD)	MDS-UPDRS III Off-medication Mean (SD)	MDS-UPDRS III On-medication Mean (SD)	MDS-UPDRS IV Mean (SD)	PDQ-39 Mean (SD)	LED Mean (SD)	MADRS Mean (SD)	NMSS Mean (SD)
Athauda et al.; 2017	**Exenatide**	31	61.6 (8.2)	22 (71%)	9.8 (4.8)	12.5 (6.7)	32.8 (9.7)	19.4 (8.4)	4.7 (3.1)	19.9 (13.7)	773.9 (260.9)	4.1 (3.7)	24.6 (19.8)
	**Placebo**	29	57.8 (8.0)	22 (76%)	9.2 (3.8)	10.7 (5.3)	27.1 (10.3)	14.4 (8.2)	5.3 (3.0)	21.1 (13.0)	825.7 (215.0)	3.7 (3.0)	28.3 (24.7)
Aviles-Olmos et al.; 2013	**Exenatide**	20	61.4 (6)	15 (75%)	10.4 (4.1)	10.2 (5.2)	31 (11.2)	23.5 (6.3)	6.3 (2.4)	19.2 (13.5)	973 (454)	10.9 (5.1)	N/A
	**Placebo**	24	59.4 (8.4)	20 (83%)	11.6 (4.7)	12.9 (6.2)	34 (16.1)	25.3 (10.7)	6.3 (3.4)	24.5 (12.8)	977 (493)	11 (5.4)	N/A
McGarry et al.; 2024	**NLY01 (5 mg)**	85	60.6 (10.0)	54 (64%)	4.0 (3.7)	5.0 (4.1)	N/A	22 (8.2)	N/A	N/A	N/A	N/A	N/A
	**NLY01 (2.5 mg)**	85	62.1 (9.0)	60 (71%)	4.2 (3.1)	4.8 (3.6)	N/A	22.7 (8.1)	N/A	N/A	N/A	N/A	N/A
	**Placebo**	84	61.8 (8.1)	52 (62%)	4.7 (4.2)	4.9 (3.6)	N/A	22.3 (9.1)	N/A	N/A	N/A	N/A	N/A
Meissner et al.; 2024	**Lixisenatide**	78	59.5 (8.1)	44 (56%)	6.1 (4)	5 (3.5)	N/A	14.8 (7.3)	0.3 (1.3)	17.4 (10.9)	317 (179)	N/A	N/A
	**Placebo**	78	59.9 (8.4)	48 (62%)	6.4 (4.2)	5.4 (4.3)	N/A	15.5 (7.8)	0.2 (0.8)	16.8 (13)	355 (215)	N/A	N/A

### Quality assessment of the included studies

**Fig. 2:**
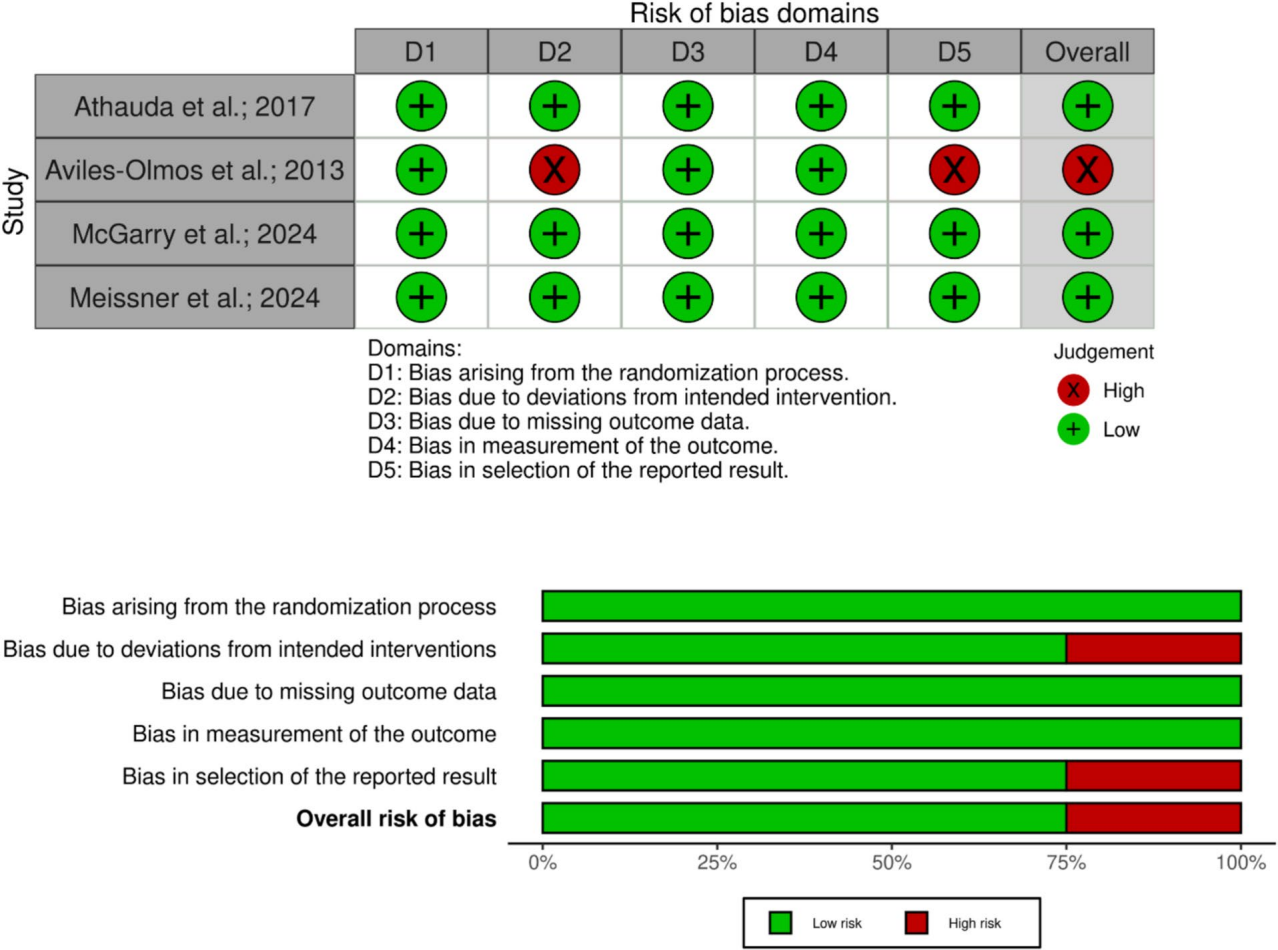
Risk of bias assessment summary

The quality of the included studies ranged from low to high risk according to the Cochrane RoB-2 tool. A summary of quality assessment domains is shown in Fig. 2. We found that the four included studies provided sufficient details regarding their randomization process and measurement of their outcomes without missing any outcome data indicating a low risk of bias in these three domains. However, the Aviles-Olmos et al. study showed both deviation from the intended intervention and selection of the reported results indicating a high risk of bias in these domains. Authors’ judgments with justifications are shown in supplementary files in Table 2.

### MDS-UPDRS III OFF-medication

**Fig. 3:**
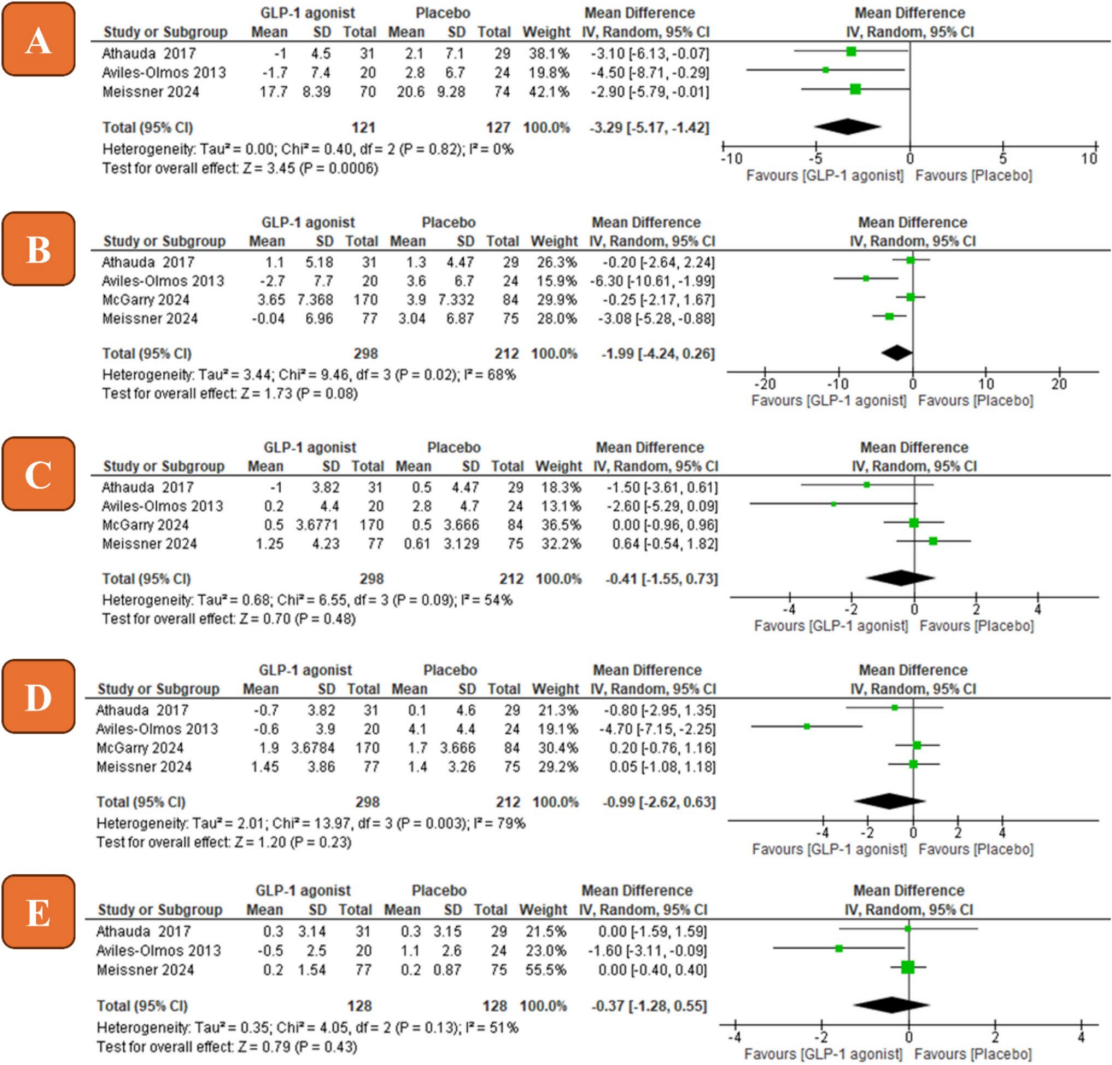
Forest plots of mean difference (*MD*) in efficacy measures (**A** MDS-UPDRS III OFF-medication; **B** MDS-UPDRS III ON-medication; **C** MDS-UPDRS I; **D** MDS-UPDRS II; **E** MDS-UPDRS IV). IV, inverse variance; CI, confidence interval

Three studies were included with a total of 248 patients. The overall mean difference between the GLP-1 agonist and the control group favored the GLP-1 agonist (MD =  − 3.29, 95% CI [− 5.17 to − 1.42], *P* = 0.0006, Fig. 3A). Pooled studies were homogenous (*P* = 0.82, *I*^2^ = 0%).

### MDS-UPDRS III ON-medication (motor function)

Four studies were included with a total of 510 patients. The overall mean difference between the GLP-1 agonist and the control group did not favor either of them (MD =  − 1.99, 95% CI [− 4.24 to 0.26], *P* = 0.08, Fig. 3B). Pooled studies were not homogenous (*P* = 0.02, *I*^2^ = 68%).

### MDS-UPDRS I (non-motor experiences of daily living)

Four studies were included with a total of 510 patients. The overall mean difference between the GLP-1 agonist and the control group did not favor either of them (MD =  − 0.41, 95% CI [− 1.55 to 0.73], *P* = 0.48, Fig. 3C). Pooled studies were not homogenous (*P* = 0.09, *I*^2^ = 54%).

### MDS-UPDRS II (motor experiences of daily living*)*

Four studies were included with a total of 510 patients. The overall mean difference between the GLP-1 agonist and the control group did not favor either of them (MD =  − 0.99, 95% CI [− 2.62 to 0.63], *P* = 0.23, Fig. 3D). Pooled studies were not homogenous (*P* = 0.003, *I*^2^ = 79%).

### MDS-UPDRS IV (motor complications)

Three studies were included with a total of 256 patients. The overall mean difference between the GLP-1 agonist and the control group did not favor either of them (MD =  − 0.37, 95% CI [− 1.28 to 0.55], *P* = 0.43, Fig. 3E). Pooled studies were homogenous (*P* = 0.13, *I*^2^ = 51%).

### Montgomery–Asberg Depression Rating Scale (MADRS)

**Fig. 4:**
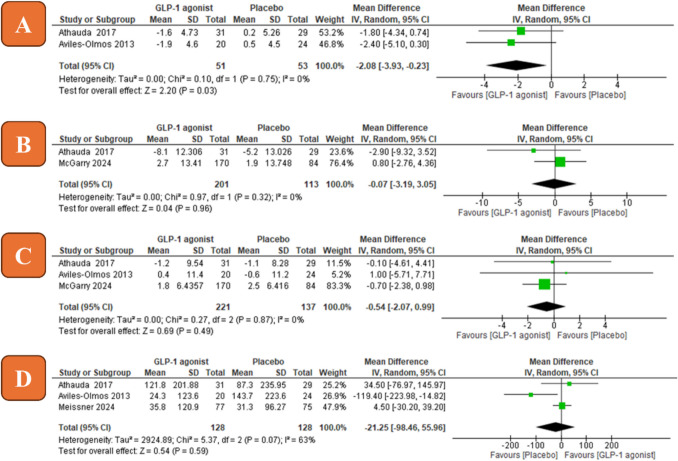
Forest plots of mean difference (*MD*) in efficacy measures (**A** MADRS; **B** NMSS; **C** PDQ-39; **D** LED). IV, inverse variance; CI, confidence interval

Two studies were included with a total of 104 patients. The overall mean difference between the GLP-1 agonist and the control group favored the GLP-1 agonist (MD =  − 2.08, 95% CI [− 3.93 to − 0.23], *P* = 0.03, Fig. 4A). Pooled studies were homogenous (*P* = 0.75, *I*^2^ = 0%).

### Non-motor Symptoms Scale (NMSS) at 9 months

Two studies were included with a total of 314 patients. The overall mean difference between the GLP-1 agonist and control group did not favor either of them (MD =  − 0.07, 95% CI [− 3.19 to 3.05], *P* = 0.96, Fig. 4B). Pooled studies were homogenous (*P* = 0.32, *I*^2^ = 0%).

### Parkinson’s Disease Questionnaire – 39 (PDQ-39)

Three studies were included with a total of 358 patients. The overall mean difference between the GLP-1 agonist and the control group did not favor either of them (MD =  − 0.54, 95% CI [− 2.07 to 0.99], *P* = 0.49, Fig. 4C). Pooled studies were homogenous (*P* = 0.87, *I*^2^ = 0%).

### L-dopa equivalent dose (LED)

Three studies were included with a total of 256 patients. The overall mean difference between the GLP-1 agonist and the control group did not favor either of them (MD =  − 21.25, 95% CI [− 98.46 to 55.96], *P* = 0.59, Fig. 4D). Pooled studies were not homogenous (*P* = 0.07, *I*^2^ = 63%).

### Safety of GLP-1 agonists on PD patients

**Fig. 5:**
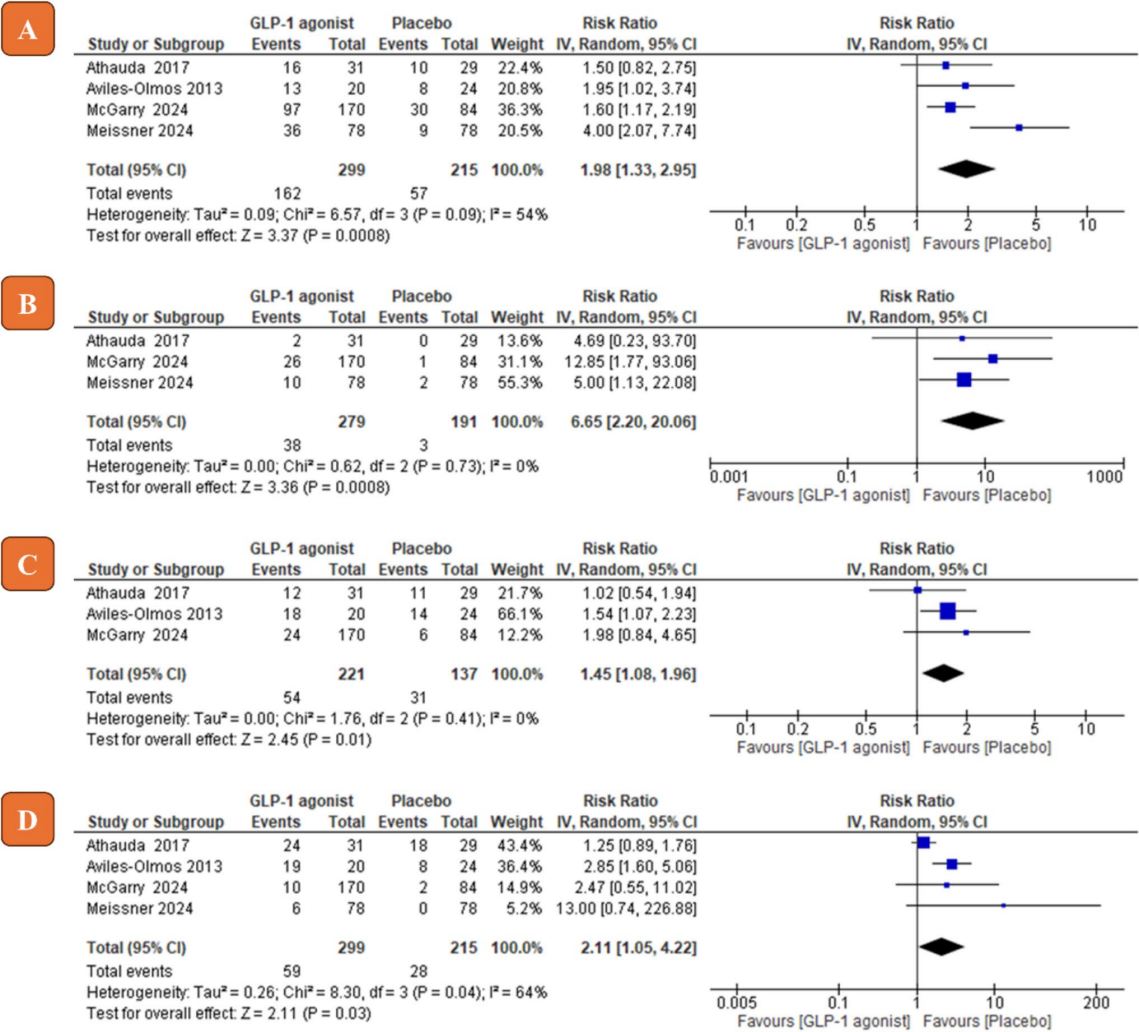
Forest plots of risk ratio (*RR*) of adverse events (**A** nausea; **B** vomiting; **C** constipation; **D** weight loss). IV, inverse variance; CI, confidence interval

**Fig. 6:**
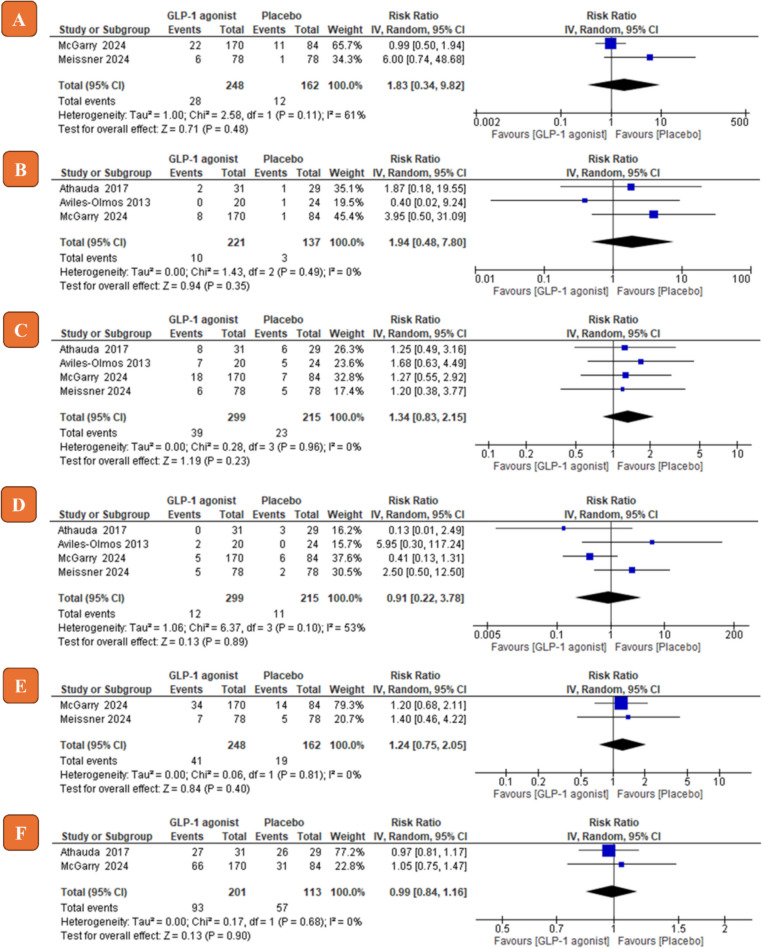
Forest plots of risk ratio (*RR*) of adverse events (**A** fatigue; **B** anxiety; **C** diarrhea; **D** UTI; **E** headache; **F** administration site disorder). IV, inverse variance; CI, confidence interval

Among the 10 adverse events analyzed, nausea, vomiting, weight loss, and constipation showed a statistically significant risk in the GLP-1 agonists group compared to the control group (nausea, RR = 1.98, 95% CI [1.33 to 2.95], *P* = 0.0008; vomiting RR = 6.65, 95% CI [2.20 to 20.06], *P* = 0.0008; weight loss RR = 2.11, 95% CI [1.05 to 4.22], *P* = 0.03; constipation RR = 1.45, 95% CI [1.08 to 1.96], *P* = 0.01; see Fig. 5). Other complications such as administration site disorder, anxiety, fatigue, headache, urinary tract infection (UTI) and diarrhea did not show a statistically significant risk in the GLP-1 agonists group compared to the control group (see Fig. 6). For all adverse events, the pooled risk ratio was homogenous (Chi-square *P* > 0.1) except for nausea and weight loss (Chi-square *P* < 0.1). Risk ratios of all adverse events with their 95% confidence intervals are shown in Table 3.

**Table 3:** Summary of the pooled risk ratios (*RR*) between the GLP-1 agonists group and control group in all reported adverse events. *RCTs* Randomized control trials. *CI* Confidence intervals

Adverse event	N of RCTs	N of patients	RR [95% CI]	Overall effect *P*-value	Heterogeneity Chi^2^ *P*-value and *I*^2^
Nausea	4	514	1.98 [1.33 to 2.95]	0.0008	*P* = 0.09, *I*^2^ = 54%
Vomiting	3	470	6.65 [2.20 to 20.6]	0.0008	*P* = 0.73, *I*^2^ = 0%
Constipation	3	358	1.45 [1.08 to 1.96]	0.01	*P* = 0.41, *I*^2^ = 0%
Weight loss	4	514	2.11 [1.05 to 4.22]	0.03	*P* = 0.04, *I*^2^ = 64%
Fatigue	2	410	1.83 [0.34 to 9.82]	0.48	*P* = 0.11, *I*^2^ = 61%
Anxiety	3	358	1.94 [0.48 to 7.80]	0.35	*P* = 0.49, *I*^2^ = 0%
Diarrhea	4	514	1.34 [0.83 to 2.15]	0.23	*P* = 0.96, *I*^2^ = 0%
UTI	4	514	0.91 [0.22 to 3.78]	0.89	*P* = 0.1, *I*^2^ = 53%
Headache	2	410	1.24 [0.75 to 2.05]	0.4	*P* = 0.81, *I*^2^ = 0%
Administration site disorder	2	314	0.99 [0.84 to 1.16]	0.9	*P* = 0.68, *I*^2^ = 0%

### Sensitivity analysis

The sensitivity analysis was conducted for efficacy outcomes that showed a significant heterogeneity (MDS-UPRDS I, MDS-UPRDS II, MDS-UPRDS III ON-medication, LED) by the exclusion method. The study of Aviles-Olmos et al. was the primary source of heterogeneity. Another meta-analysis conducted after the exclusion of the study caused the heterogeneity resulting in a significant decrease in the heterogeneity of MDS-UPDRS I (*P* = 0.22, *I*^2^ = 34%), MDS-UPDRS II (*P* = 0.71, *I*^2^ = 0%), MDS-UPDRS III ON-medication (*P* = 0.11, *I*^2^ = 54%), LED (*P* = 0.61, *I*^2^ = 0%). The overall mean difference with 95% CI between the GLP-1 agonists group and control group after sensitivity analysis did not favor either of them (MDS-UPDRS I, MD =  − 0.0, CI [− 0.92 to − 0.230.92], *P* = 1; MDS-UPDRS II, MD = 0.04, CI [− 0.65 to 0.73], *P* = 0.91; MDS-UPDRS III ON-medication, MD =  − 1.17, CI [− 3.03 to 0.69], *P* = 0.22; LED, MD = 7.15, CI [− 25.98 to 40.28], *P* = 0.67).

Weight loss and nausea are the only two heterogeneous adverse events. A sensitivity analysis was performed by excluding each study independently from the meta-analysis. For weight loss, the heterogeneity was resolved by removing either Athauda et al. study (*P* = 0.58, *I*^2^ = 0%) or Aviles-Olmos et al. study (*P* = 0.20, *I*^2^ = 38%) with a more significant decrease in heterogeneity in the case of excluding Athauda et al. study. For nausea, the heterogeneity was significantly resolved by excluding Meissner et al. study (*P* = 0.83, *I*^2^ = 0%). Sensitivity analysis of all heterogeneous outcomes is shown in the supplementary materials.

A sensitivity analysis was conducted including a pre-printed study by Hogg et al. exploring the effect of adding it to the analysis which resulted in the meta-analysis of UPRDS-III OFF-medication to become insignificant (MD =  − 2.19, 95% CI [− 4.77 to 0.38], *P* = 0.1) while all other analysis remained the same (see the forest plots from Fig. 8 to Fig. 23 in the supplementary materials).

## Discussion

We found that GLP-1 agonists significantly improve the motor functions of PD patients with no significant improvement in their mood or quality of life. In terms of safety, the analyzed data showed the risk of nausea, vomiting, constipation, and weight loss in the GLP-1 agonists group compared to the control group.

The studies included in our analysis employed different forms and doses of GLP-1 agonists which may contribute to the variability found among the pooled effect sizes reported by our study. Athauda et al. study used exenatide 2 mg administered as a once-weekly subcutaneous injection for 48 weeks then 12 weeks washout period (Athauda et al. 2017). Patients in the experimental group of Aviles-Olmos et al. study received exenatide 5 µg as a twice-daily injection during the first month followed by exenatide 10 µg twice-daily injection for the subsequent 11 months followed by 2 months of washout period (Aviles-Olmos et al. 2013). Meissner et al. utilized another form of GLP-1 agonist, Lixisenatide, which has a high affinity for GLP-1 receptors with an initial dose of 10 µg per day for 2 weeks, then the dose increased to 20 µg per day for the rest of the trial, which is 12 months followed by 2 months of washout period, administered via subcutaneous injection (Meissner et al. 2024). McGarry et al. study used NLY01, a longer-lasting version of exenatide, administered subcutaneously once a week at doses of either 2.5 mg or 5.0 mg for 36 weeks without subsequent washout period (McGarry et al. 2024). The preprinted study used a more potent GLP-1 agonist, liraglutide, administered as 1.2 or 1.8 mg one-daily; however, we did not include it in our main meta-analysis for fear of conflicting information between the preprinted form and the future published form of the paper (Brietzke et al. 2023; Hogg et al. 2022). Another recent study by Nelson et al. (2022) suggests that preprints can contribute to decision-making, so with all this evidence, we decided to conduct another meta-analysis that included the preprinted study (shown in supplementary material) without adding it to our main results or discussion.

In terms of MDS-UPDRS III ON-medication, data showed no statistically significant difference between the GLP-1 agonists group and the control group (MD =  − 1.99, *P* = 0.08). In the case of OFF-medication state, however, the data is statistically higher in the GLP-1 agonists group compared to the control group (MD =  − 3.29, *P* = 0.0006) with no significant heterogeneity (*P* = 0.82, *I*^2^ = 0%). The − 3.29 exceeds the minimal clinically important difference (MCID) for improvement in the motor impairment of − 3.25 calculated by Horváth et al. (2015). These findings are aligned with the adjusted data in the previously conducted systematic review by Mulvaney et al. (2020). In case of motor complications assessed by MDS-UPDRS IV, there is no significant difference between the GLP-1 agonists group and the control group, and the data were homogenous.

The mental and behavioral mood of PD patients was evaluated using MDS-UPDRS I. There was no statistically significant difference between the GLP-1 agonists group compared to the control group which aligned with the previous systematic review made by Mulvaney et al. (2020). There was a significant heterogeneity in this outcome which was resolved by excluding the Aviles-Olmos et al. study during the sensitivity analysis. The possible reason for this heterogeneity is Aviles-Olmos et al. study used a very small dose of exenatide compared to the other studies. Another reason could be the small sample size of the Aviles-Olmos et al. study as it is the least sample size of the four included RCTs or could be the difference in the stage of PD each study is dealing with. In most adverse events, there was no significant heterogeneity between studies; however, significant heterogeneity was observed in nausea and weight loss. Heterogeneity in nausea was resolved by excluding the Meissner et al. study as it experimented with Lixisenatide which had an association with less nausea compared to other GLP-1 agonists (Bettge et al. 2017). Heterogeneity in weight loss could be resolved by excluding Aviles-Olmos et al. study as its population was moderate Parkinson’s patients where weight changes were associated with disease severity compared with other studies (Ghourchian et al. 2021).

GLP-1 agonists recently showed positive results as an antidepressant. A recent systematic review conducted on 2701 depressed patients showed a decrease in the depression rates of the group that received GLP-1 agonists compared to the control group (X. Chen et al. 2024). These findings aligned with our findings as PD patients receiving GLP-1 agonists showed a statistically significant improvement on MADRS scale compared to those in the control group. In terms of quality of life, there was no statistically significant difference between the GLP-1 agonists group and the control group. The mean difference of PDQ-39 (MD =  − 0.54, *P* = 0.49) did not exceed the minimal clinically important difference (MCID) for improvement of quality of life of − 4.72 calculated by Horváth et al. (2015).

Both Athauda et al. and McGarry et al. studies assess the NMSS score at 6, 9, 12, and 15 months or 9 months only respectively, so we decided to evaluate NMSS at 9 months only aiming to decrease the heterogeneity. There was no statistically significant difference between the GLP-1 agonists group and the control group and there was no significant heterogeneity between the two studies. The high weight of the McGarry et al. study (76.4%) is due to the large sample size compared to the other included study due to the combination of the two intervention groups as we mentioned before.

Most studies investigating GLP-1 receptors, whether as a drug for type 2 diabetes or obesity, have shown that the most common adverse events were gastrointestinal (GI) events (Dungan et al. 2014; Pratley et al. 2014; Rosenstock et al. 2013), which is consistent with our findings. These events are due to the effect of GLP-1 agonists as they slow gastric emptying by activating the vagus nerve and activating central appetite suppression (Filippatos et al. 2014; Zheng et al. 2024). This may be a serious problem for Parkinson’s patients as they already have GI problems (Skjærbæk et al. 2021). The prevalence of constipation in PD patients is approximately 50% (H. Chen et al. 2015). Some studies suggest that constipation begins 20 years before the onset of motor complications of PD (Savica et al. 2009). Our findings show that GLP-1 agonists have a high risk of constipation among PD patients taking GLP-1 agonists. Probiotics, another newly suggested treatment for PD, may help manage constipation in PD patients as they were evident to help with constipation (Xie et al. 2023; Zeng et al. 2023). However, a recent systematic review suggests that increasing the number of prescribed drugs for PD patients could aggravate their psychological problems (Bhagavathula et al. 2022). Our results also showed that GLP-1 agonists had a higher risk of weight loss compared to the control group, which is a serious problem that most Parkinson’s patients suffer from (Ma et al. 2018). A previous study reported that the prevalence of weight loss among PD patients is about 48.6% (Cersosimo et al. 2018). This finding should be considered because weight loss can affect the quality of life of PD patients (Akbar et al. 2015). This may be one of the reasons why GLP-1 agonists did not significantly improve the quality of life of PD patients.

The GI side effects of GLP-1 receptors may lead to a significant placebo effect bias in the GLP-1 agonists group compared to the control group. GLP-1 agonists as medications for type 2 diabetes affect the GI system, leading PD to expect an improvement in their condition because they feel that the medication is working inside their body, in the form of GI symptoms. These expectations can psychologically influence Parkinson’s patients, biasing subjective scores such as the NMSS, MADRS, and PDQ-39, as patients self-report their scores, making the results from these scales less reliable to consider; however, this can also occur in semi-objective scales such as the MDS-UPDRS, even though rely on clinician-rated assessment and patient-reported scores. A systematic review by Quattrone et al. showed that a placebo effect can lead to motor improvement in Parkinson’s patients (Quattrone et al. 2018). This may be due to the strength of belief in improvement that can regulate dopamine release in PD from all areas of the striatum (Lidstone et al. 2010). All this could be due to a placebo effect induced by GI symptoms; however, this could be mitigated by future studies comparing GLP-1 agonists with other medications.

## Limitations and recommendations

Despite all our included studies being RCTs, comparing GLP-1 agonists to placebo or conventional treatment, some limitations should be considered. There was a significant heterogeneity in terms of MDS-UPDRS I, II, III ON-medication and LED due to different reasons ranging from differences in the dosage treatment to the large variation found in the sample size of the four included RCTs. Another limitation is the lack of focus of the RCTs on a specific PD stage. These all limit the generalizability of our findings and identify which stage of PD would benefit the most from the medication. Thus, we recommend putting standardized protocols to facilitate future comparisons between studies. We also recommend further studies on other forms of GLP-1 agonists rather than exenatide and its forms to evaluate each drug individually and know its efficacy in each PD stage. We also recommend using objective rather than subjective measuring methods to decrease the placebo effect bias in the treatment group results from the GI symptoms of GLP-1 agonists.

By addressing these limitations and considering these recommendations, future research studies would be more beneficial and effective in evaluating the effect of different forms of GLP-1 agonists on specific categories of PD patients.

## Conclusion

In conclusion, GLP-1 agonists could be efficient in improving the motor function of PD patients. However, current evidence is still insufficient to prove these findings as well as assess the mood and quality of life in PD patients. Therefore, further studies are needed with standardized protocols, a larger sample size, and a specific focus on a PD stage.

## Supplementary Information

Below is the link to the electronic supplementary material.

Supplementary file1 (DOCX 272 KB)

## Data Availability

All source data for this work (or generated in this study) are available upon reasonable request.

## Abbreviations

*GLP-1*: Glucagon-like peptide – 1
*LED*: L-dopa equivalent dose
*MADRS*: Montgomery-Asberg Depression Rating Scale
*MDS-UPDRS*: Movement Disorder Society – Unified Parkinson Disease Rating Scale
*NMSS*: Non-Motor Symptoms Scale
*PD*: Parkinson’s disease
*PDQ-39*: Parkinson’s disease Questionnaire – 39

## References

[CR1] Abbas A, Hefnawy MT, Negida A (2024) Meta-analysis accelerator: a comprehensive tool for statistical data conversion in systematic reviews with meta-analysis. BMC Med Res Methodol 24(1):243. 10.1186/s12874-024-02356-6 39425031 10.1186/s12874-024-02356-6 PMC11487830

[CR2] Akbar U, He Y, Dai Y, Hack N, Malaty I, McFarland NR, Hess C, Schmidt P, Wu S, Okun MS (2015) Weight loss and impact on quality of life in Parkinson’s disease. PLoS ONE 10(5):e0124541. 10.1371/journal.pone.0124541 25938478 10.1371/journal.pone.0124541 PMC4418600

[CR3] Aksoy D, Solmaz V, Çavuşoğlu T, Meral A, Ateş U, Erbaş O (2017) Neuroprotective effects of exenatide in a rotenone-induced rat model of Parkinson’s disease. Am J Med Sci 354(3):319–324. 10.1016/j.amjms.2017.05.002 28918840 10.1016/j.amjms.2017.05.002

[CR4] Athauda D, Maclagan K, Skene SS, Bajwa-Joseph M, Letchford D, Chowdhury K, Hibbert S, Budnik N, Zampedri L, Dickson J, Li Y, Aviles-Olmos I, Warner TT, Limousin P, Lees AJ, Greig NH, Tebbs S, Foltynie T (2017) Exenatide once weekly versus placebo in Parkinson’s disease: a randomised, double-blind, placebo-controlled trial. Lancet 390(10103):1664–1675. 10.1016/S0140-6736(17)31585-4 28781108 10.1016/S0140-6736(17)31585-4 PMC5831666

[CR5] Aviles-Olmos I, Dickson J, Kefalopoulou Z, Djamshidian A, Ell P, Soderlund T, Whitton P, Wyse R, Isaacs T, Lees A, Limousin P, Foltynie T (2013) Exenatide and the treatment of patients with Parkinson’s disease. J Clin Investig 123(6):2730–2736. 10.1172/JCI68295 23728174 10.1172/JCI68295 PMC3668846

[CR6] Bettge K, Kahle M, Abd El Aziz MS, Meier JJ, Nauck MA (2017) Occurrence of nausea, vomiting and diarrhoea reported as adverse events in clinical trials studying glucagon-like peptide-1 receptor agonists: a systematic analysis of published clinical trials. Diabetes Obes Metab 19(3):336–347. 10.1111/dom.12824 27860132 10.1111/dom.12824

[CR7] Bhagavathula AS, Tesfaye W, Vidyasagar K, Fialova D (2022) Polypharmacy and hyperpolypharmacy in older individuals with Parkinson’s disease: a systematic review and meta-analysis. Gerontology 68(10):1081–1090. 10.1159/000521214 35026767 10.1159/000521214 PMC9677850

[CR8] Brietzke E, Gomes FA, Gerchman F, Freire RCR (2023) Should systematic reviews and meta-analyses include data from preprints? Trends Psychiatry Psychother 45:e20210324. 10.47626/2237-6089-2021-0324 10.47626/2237-6089-2021-0324 PMC10164401 34551239

[CR9] Cersosimo MG, Raina GB, Pellene LA, Micheli FE, Calandra CR, Maiola R (2018) Weight loss in Parkinson’s disease: the relationship with motor symptoms and disease progression. Biomed Res Int 2018:9642524. 10.1155/2018/9642524 30105269 10.1155/2018/9642524 PMC6076942

[CR10] Chang MH, Chang TW, Lai PH, Sy CG (1995) Resting tremor only: a variant of Parkinson’s disease or of essential tremor. J Neurol Sci 130(2):215–219. 10.1016/0022-510x(95)00033-x 8586989 10.1016/0022-510x(95)00033-x

[CR11] Chaudhuri KR, Martinez-Martin P, Brown RG, Sethi K, Stocchi F, Odin P, Ondo W, Abe K, Macphee G, Macmahon D, Barone P, Rabey M, Forbes A, Breen K, Tluk S, Naidu Y, Olanow W, Williams AJ, Thomas S, Schapira AHV (2007) The metric properties of a novel non-motor symptoms scale for Parkinson’s disease: results from an international pilot study. Mov Disord 22(13):1901–1911. 10.1002/mds.21596 17674410 10.1002/mds.21596

[CR12] Chen H, Zhao EJ, Zhang W, Lu Y, Liu R, Huang X, Ciesielski-Jones AJ, Justice MA, Cousins DS, Peddada S (2015) Meta-analyses on prevalence of selected Parkinson’s nonmotor symptoms before and after diagnosis. Transl Neurodegener 4(1):1. 10.1186/2047-9158-4-1 25671103 10.1186/2047-9158-4-1 PMC4322463

[CR13] Chen X, Zhao P, Wang W, Guo L, Pan Q (2024) The antidepressant effects of GLP-1 receptor agonists: a systematic review and meta-analysis. Am J Geriatr Psychiatry 32(1):117–127. 10.1016/j.jagp.2023.08.010 37684186 10.1016/j.jagp.2023.08.010

[CR14] Collins L, Costello RA (2024) Glucagon-like peptide-1 receptor agonists. In *StatPearls*. StatPearls Publishing. http://www.ncbi.nlm.nih.gov/books/NBK551568/31855395

[CR15] Dauer W, Przedborski S (2003) Parkinson’s disease: mechanisms and models. Neuron 39(6):889–909. 10.1016/S0896-6273(03)00568-3 12971891 10.1016/s0896-6273(03)00568-3

[CR16] Dorsey ER, Sherer T, Okun MS, Bloem BR, Brundin P, Langston JW, Bloem BR (2018) The emerging evidence of the Parkinson pandemic. J Parkinsons Dis 8(s1):S3–S8. 10.3233/JPD-181474 30584159 10.3233/JPD-181474 PMC6311367

[CR17] Dungan KM, Povedano ST, Forst T, González JGG, Atisso C, Sealls W, Fahrbach JL (2014) Once-weekly dulaglutide versus once-daily liraglutide in metformin-treated patients with type 2 diabetes (AWARD-6): a randomised, open-label, phase 3, non-inferiority trial. Lancet 384(9951):1349–1357. 10.1016/S0140-6736(14)60976-4 25018121 10.1016/S0140-6736(14)60976-4

[CR18] Egger M, Smith GD, Schneider M, Minder C (1997) Bias in meta-analysis detected by a simple, graphical test. BMJ 315(7109):629–634. 10.1136/bmj.315.7109.629 9310563 10.1136/bmj.315.7109.629 PMC2127453

[CR19] Elbassuoni EA, Ahmed RF (2019) Mechanism of the neuroprotective effect of GLP-1 in a rat model of Parkinson’s with pre-existing diabetes. Neurochem Int 131:104583. 10.1016/j.neuint.2019.104583 31654678 10.1016/j.neuint.2019.104583

[CR20] Filippatos TD, Panagiotopoulou TV, Elisaf MS (2014) Adverse effects of GLP-1 receptor agonists. Rev Diabet Stud: RDS 11(3–4):202–230. 10.1900/RDS.2014.11.202 26177483 10.1900/RDS.2014.11.202 PMC5397288

[CR21] Fritsch T, Smyth KA, Wallendal MS, Hyde T, Leo G, Geldmacher DS (2012) Parkinson disease: research update and clinical management. South Med J 105(12):650–656. 10.1097/SMJ.0b013e318273a60d 23211499 10.1097/SMJ.0b013e318273a60d

[CR22] Ghourchian S, Gruber-Baldini AL, Shakya S, Herndon J, Reich SG, von Coelln R, Savitt JM, Shulman LM (2021) Weight loss and weight gain in Parkinson disease. Parkinsonism Relat Disord 83:31–36. 10.1016/j.parkreldis.2020.12.018 33465545 10.1016/j.parkreldis.2020.12.018

[CR23] Gilman CP, Perry T, Furukawa K, Grieg NH, Egan JM, Mattson MP (2003) Glucagon-like peptide 1 modulates calcium responses to glutamate and membrane depolarization in hippocampal neurons. J Neurochem 87(5):1137–1144. 10.1046/j.1471-4159.2003.02073.x 14622093 10.1046/j.1471-4159.2003.02073.x

[CR24] Goetz CG, Tilley BC, Shaftman SR, Stebbins GT, Fahn S, Martinez-Martin P, Poewe W, Sampaio C, Stern MB, Dodel R, Dubois B, Holloway R, Jankovic J, Kulisevsky J, Lang AE, Lees A, Leurgans S, LeWitt PA, Nyenhuis D, … Movement Disorder Society UPDRS Revision Task Force (2008) Movement Disorder Society-sponsored revision of the Unified Parkinson’s Disease Rating Scale (MDS-UPDRS): scale presentation and clinimetric testing results. Mov Disord 23(15), 2129–2170. 10.1002/mds.22340 10.1002/mds.22340 19025984

[CR25] Higgins JP, Eldridge S, Li T (2019a) Including variants on randomized trials. In: Cochrane Handbook for systematic reviews of interventions. John Wiley & Sons, Ltd., pp 569–593. 10.1002/9781119536604.ch23

[CR26] Higgins JP, Li T, Deeks JJ (2019b) Choosing effect measures and computing estimates of effect. In: Cochrane handbook for systematic reviews of interventions. John Wiley & Sons, Ltd., pp 143–176. 10.1002/9781119536604.ch6

[CR27] Higgins JP, Savović J, Page MJ, Elbers RG, Sterne JA (2019c) Assessing risk of bias in a randomized trial. In: Cochrane handbook for systematic reviews of interventions. John Wiley & Sons, Ltd., pp. 205–228. 10.1002/9781119536604.ch8

[CR28] Higgins JPT, Thomas J, Chandler J, Cumpston M, Li T, Page MJ, Welch VA (2019d) Cochrane handbook for systematic reviews of interventions. 10.1002/9781119536604 10.1002/14651858.ED000142 PMC10284251 31643080

[CR29] Hogg E, Wu T, Bresee C, Wertheimer J, Malatt C, Tan E, Pomeroy H, Nuno M, Wyse R, Tagliati M (2022). A phase II, Randomized, double-blinded, placebo-controlled trial of liraglutide in Parkinson’s disease (SSRN Scholarly Paper No. 4212371). Social Science Research Network. 10.2139/ssrn.4212371

[CR30] Horváth K, Aschermann Z, Ács P, Deli G, Janszky J, Komoly S, Balázs É, Takács K, Karádi K, Kovács N (2015) Minimal clinically important difference on the motor examination part of MDS-UPDRS. Parkinsonism Relat Disord 21(12):1421–1426. 10.1016/j.parkreldis.2015.10.006 26578041 10.1016/j.parkreldis.2015.10.006

[CR31] Jenkinson C, Fitzpatrick R, Peto V, Greenhall R, Hyman N (1997) The Parkinson’s Disease Questionnaire (PDQ-39): development and validation of a Parkinson’s disease summary index score. Age Ageing 26(5):353–357. 10.1093/ageing/26.5.353 9351479 10.1093/ageing/26.5.353

[CR32] Kim J-S, Sung H-Y (2015). Gastrointestinal autonomic dysfunction in patients with Parkinson’s disease. J Mov Disord 8(2):76–82. 10.14802/jmd.15008 10.14802/jmd.15008 PMC4460543 26090079

[CR33] Li Y, Perry T, Kindy MS, Harvey BK, Tweedie D, Holloway HW, Powers K, Shen H, Egan JM, Sambamurti K, Brossi A, Lahiri DK, Mattson MP, Hoffer BJ, Wang Y, Greig NH (2009) GLP-1 receptor stimulation preserves primary cortical and dopaminergic neurons in cellular and rodent models of stroke and Parkinsonism. Proc Natl Acad Sci 106(4):1285–1290. 10.1073/pnas.0806720106 19164583 10.1073/pnas.0806720106 PMC2633544

[CR34] Lidstone SC, Schulzer M, Dinelle K, Mak E, Sossi V, Ruth TJ, de la Fuente-Fernández R, Phillips AG, Stoessl AJ (2010) Effects of expectation on placebo-induced dopamine release in Parkinson disease. Arch Gen Psychiatry 67(8):857–865. 10.1001/archgenpsychiatry.2010.88 20679593 10.1001/archgenpsychiatry.2010.88

[CR35] Löhle M, Storch A, Reichmann H (2009) Beyond tremor and rigidity: non-motor features of Parkinson’s disease. J Neural Transm 116(11):1483–1492. 10.1007/s00702-009-0274-1 19680598 10.1007/s00702-009-0274-1

[CR36] Ma K, Xiong N, Shen Y, Han C, Liu L, Zhang G, Wang L, Guo S, Guo X, Xia Y, Wan F, Huang J, Lin Z, Wang T (2018) Weight loss and malnutrition in patients with Parkinson’s disease: current knowledge and future prospects. Front Aging Neurosci 10:1. 10.3389/fnagi.2018.00001 29403371 10.3389/fnagi.2018.00001 PMC5780404

[CR37] McGarry A, Rosanbalm S, Leinonen M, Olanow CW, To D, Bell A, Lee D, Chang J, Dubow J, Dhall R, Burdick D, Parashos S, Feuerstein J, Quinn J, Pahwa R, Afshari M, Ramirez-Zamora A, Chou K, Tarakad A, … Kieburtz K (2024) Safety, tolerability, and efficacy of NLY01 in early untreated Parkinson’s disease: a randomised, double-blind, placebo-controlled trial. Lancet Neurol 23(1):37–45. 10.1016/S1474-4422(23)00378-2 10.1016/S1474-4422(23)00378-2 38101901

[CR38] McGuinness LA, Higgins JPT (2021) Risk-of-bias VISualization (robvis): an R package and Shiny web app for visualizing risk-of-bias assessments. Research Synthesis Methods 12(1):55–61. 10.1002/jrsm.1411 32336025 10.1002/jrsm.1411

[CR39] Meissner, W. G., Remy, P., Giordana, C., Maltête, D., Derkinderen, P., Houéto, J.-L., Anheim, M., Benatru, I., Boraud, T., Brefel-Courbon, C., Carrière, N., Catala, H., Colin, O., Corvol, J.-C., Damier, P., Dellapina, E., Devos, D., Drapier, S., Fabbri, M., … Rascol, O. (2024). Trial of lixisenatide in early Parkinson’s disease. *New England Journal of Medicine*, *390*(13), 1176–1185. 10.1056/NEJMoa2312323 10.1056/NEJMoa2312323 38598572

[CR40] Mhyre TR, Boyd JT, Hamill RW, Maguire-Zeiss KA (2012) Parkinson’s disease. In: Harris JR (Ed.), Protein Aggregation and Fibrillogenesis in Cerebral and Systemic Amyloid Disease (pp. 389–455). Springer Netherlands. 10.1007/978-94-007-5416-4_16

[CR41] Montgomery SA, Asberg M (1979) A new depression scale designed to be sensitive to change. Br J Psychiatry 134:382–389. 10.1192/bjp.134.4.382 444788 10.1192/bjp.134.4.382

[CR42] Moustafa AA, Chakravarthy S, Phillips JR, Gupta A, Keri S, Polner B, Frank MJ, Jahanshahi M (2016) Motor symptoms in Parkinson’s disease: a unified framework. Neurosci Biobehav Rev 68:727–740. 10.1016/j.neubiorev.2016.07.010 27422450 10.1016/j.neubiorev.2016.07.010

[CR43] Mulvaney CA, Duarte GS, Handley J, Evans DJ, Menon S, Wyse R, Emsley HC (2020) GLP‐1 receptor agonists for Parkinson’s disease—Mulvaney, CA - 2020 | Cochrane Library. https://www.cochranelibrary.com/cdsr/doi/10.1002/14651858.CD012990.pub2/full 10.1002/14651858.CD012990.pub2 PMC7390475 32700772

[CR44] Nelson L, Ye H, Schwenn A, Lee S, Arabi S, Hutchins BI (2022) Robustness of evidence reported in preprints during peer review. Lancet Glob Health 10(11):e1684–e1687. 10.1016/S2214-109X(22)00368-0 36240832 10.1016/S2214-109X(22)00368-0 PMC9553196

[CR45] Ouzzani M, Hammady H, Fedorowicz Z, Elmagarmid A (2016) Rayyan—a web and mobile app for systematic reviews. Syst Rev 5(1):210. 10.1186/s13643-016-0384-4 27919275 10.1186/s13643-016-0384-4 PMC5139140

[CR46] Page MJ, McKenzie JE, Bossuyt PM, Boutron I, Hoffmann TC, Mulrow CD, Shamseer L, Tetzlaff JM, Akl EA, Brennan SE, Chou R, Glanville J, Grimshaw JM, Hróbjartsson A, Lalu MM, Li T, Loder EW, Mayo-Wilson E, McDonald S, … Moher D (2021) The PRISMA 2020 statement: an updated guideline for reporting systematic reviews. BMJ372:n71. 10.1136/bmj.n71 10.1136/bmj.n71 PMC8005924 33782057

[CR47] Perry T, Haughey NJ, Mattson MP, Egan JM, Greig NH (2002a) Protection and reversal of excitotoxic neuronal damage by glucagon-like peptide-1 and exendin-4. J Pharmacol Exp Ther 302(3):881–888. 10.1124/jpet.102.037481 12183643 10.1124/jpet.102.037481

[CR48] Perry T, Lahiri DK, Chen D, Zhou J, Shaw KTY, Egan JM, Greig NH (2002b) A novel neurotrophic property of glucagon-like peptide 1: a promoter of nerve growth factor-mediated differentiation in PC12 Cells. J Pharmacol Exp Ther 300(3):958–966. 10.1124/jpet.300.3.958 11861804 10.1124/jpet.300.3.958

[CR49] Perry T, Lahiri DK, Sambamurti K, Chen D, Mattson MP, Egan JM, Greig NH (2003) Glucagon-like peptide-1 decreases endogenous amyloid-β peptide (Aβ) levels and protects hippocampal neurons from death induced by Aβ and iron. J Neurosci Res 72(5):603–612. 10.1002/jnr.10611 12749025 10.1002/jnr.10611

[CR50] Pratley RE, Nauck MA, Barnett AH, Feinglos MN, Ovalle F, Harman-Boehm I, Ye J, Scott R, Johnson S, Stewart M, Rosenstock J (2014) Once-weekly albiglutide versus once-daily liraglutide in patients with type 2 diabetes inadequately controlled on oral drugs (HARMONY 7): a randomised, open-label, multicentre, non-inferiority phase 3 study. Lancet Diabetes Endocrinol 2(4):289–297. 10.1016/S2213-8587(13)70214-6 24703047 10.1016/S2213-8587(13)70214-6

[CR51] Quattrone A, Barbagallo G, Cerasa A, Stoessl AJ (2018) Neurobiology of placebo effect in Parkinson’s disease: what we have learned and where we are going. Mov Disord 33(8):1213–1227. 10.1002/mds.27438 30230624 10.1002/mds.27438

[CR52] Rosenstock J, Raccah D, Korányi L, Maffei L, Boka G, Miossec P, Gerich JE (2013) Efficacy and safety of lixisenatide once daily versus exenatide twice daily in type 2 diabetes inadequately controlled on metformin: a 24-week, randomized, open-label, active-controlled study (GetGoal-X). Diabetes Care 36(10):2945–2951. 10.2337/dc12-2709 23698396 10.2337/dc12-2709 PMC3781502

[CR53] Savica R, Carlin JM, Grossardt BR, Bower JH, Ahlskog JE, Maraganore DM, Bharucha AE, Rocca WA (2009) Medical records documentation of constipation preceding Parkinson disease. Neurology 73(21):1752–1758. 10.1212/WNL.0b013e3181c34af5 19933976 10.1212/WNL.0b013e3181c34af5 PMC2788809

[CR54] Skjærbæk C, Knudsen K, Horsager J, Borghammer P (2021). Gastrointestinal dysfunction in Parkinson’s disease. J Clin Med 10(3):3. 10.3390/jcm10030493 10.3390/jcm10030493 PMC7866791 33572547

[CR55] Vilsbøll T, Christensen M, Junker AE, Knop FK, Gluud LL (2012) Effects of glucagon-like peptide-1 receptor agonists on weight loss: systematic review and meta-analyses of randomised controlled trials. BMJ 344:d7771. 10.1136/bmj.d7771 22236411 10.1136/bmj.d7771 PMC3256253

[CR56] Xie L, Chen D, Zhu X, Cheng C (2023) Efficacy and safety of probiotics in Parkinson’s constipation: a systematic review and meta-analysis. Front Pharmacol 13. 10.3389/fphar.2022.1007654 10.3389/fphar.2022.1007654 PMC9871263 36703760

[CR57] Zafar S, Yaddanapudi SS (2023) Parkinson disease. In: *StatPearls*. StatPearls Publishing. http://www.ncbi.nlm.nih.gov/books/NBK470193/29261972

[CR58] Zeng Y, Wu Y, Zhang Q, Xiao X (2023) Crosstalk between glucagon-like peptide 1 and gut microbiota in metabolic diseases. mBio 15(1):e02032-23. 10.1128/mbio.02032-23 38055342 10.1128/mbio.02032-23 PMC10790698

[CR59] Zheng Z, Zong Y, Ma Y, Tian Y, Pang Y, Zhang C, Gao J (2024) Glucagon-like peptide-1 receptor: mechanisms and advances in therapy. Signal Transduct Target Ther 9(1):1–29. 10.1038/s41392-024-01931-z 39289339 10.1038/s41392-024-01931-z PMC11408715

