# Supplementary material for: Efficacy and safety of GLP-1 agonists in Parkinson’s disease: a systematic review and meta-analysis of randomized controlled trials
Source: Naunyn Schmiedebergs Arch Pharmacol. 2025 Mar 11;398(8):9721–36. doi: 10.1007/s00210-025-03932-3 (PMC12350457; doi:10.1007/s00210-025-03932-3)
Supplement: Supplementary file 1 — Supplementary file1 (DOCX 272 KB) [file 210_2025_3932_MOESM1_ESM.docx]

**Supplementary Materials**

[**Tables 3**](#_Toc185093588)

[Table 1 Search strategy 3](#_Toc185093589)

[Table 2.1 Authors judgment with justifications of Athauda et al.; 2017 5](#_Toc185093590)

[Table 2.2 Authors judgment with justifications of Aviles-Olmos et al.; 2013 6](#_Toc185093591)

[Table 2.3 Authors judgment with justifications of McGarry et al.; 2024 7](#_Toc185093592)

[Table 2.4 Authors judgment with justifications of Meissner et al.; 2024 8](#_Toc185093593)

[Table 2.5 Authors judgment with justifications of Hogg et al.; preprinted 9](#_Toc185093594)

[**Sensitivity analysis forest plots 10**](#_Toc185093595)

[Figure 1 Sensitivity analysis of MDS-UPDRS I 10](#_Toc185093596)

[Figure 2 Sensitivity analysis of MDS-UPDRS II 10](#_Toc185093597)

[Figure 3 Sensitivity analysis of MDS-UPDRS III on-medication 10](#_Toc185093598)

[Figure 4 Sensitivity analysis of LED 10](#_Toc185093599)

[Figure 5 Sensitivity analysis of Nausea 11](#_Toc185093600)

[Figure 6.1 Sensitivity analysis of weight loss (excluding Aviles-Olmos et al.; 2013) 11](#_Toc185093601)

[Figure 5.2 Sensitivity analysis of weight loss (excluding Athauda et al.; 2017) 11](#_Toc185093602)

[Figure 7.1 Sensitivity analysis of UTI (excluding McGarry et al.; 2024) 11](#_Toc185093603)

[Figure 6.2 Sensitivity analysis of UTI (excluding Meissner et al.; 2024) 12](#_Toc185093604)

[**Meta-analysis including the pre-printed paper 13**](#_Toc185093605)

[Figure 8 Forest plot of MDS-UPDRS I including Hogg et al.; pre-printed 13](#_Toc185093606)

[Figure 9 Forest plot of MDS-UPRDS II including Hogg et al.; pre-printed 13](#_Toc185093607)

[Figure 10 Forest plot of MDS-UPDRS III On-medication including Hogg et al.; pre-printed 13](#_Toc185093608)

[Figure 11 Forest plot of MDS-UPRDS III Off-medication including Hogg et al.; pre-printed 13](#_Toc185093609)

[Figure 12 Forest plot of MDS-UPRDS IV including Hogg et al.; pre-printed 14](#_Toc185093610)

[Figure 13 Forest plot of PDQ-39 including Hogg et al.; pre-printed 14](#_Toc185093611)

[Figure 14 Forest plot of NMSS including Hogg et al.; pre-printed 14](#_Toc185093612)

[Figure 15 Forest plot of Administration site disorder including Hogg et al.; pre-printed 14](#_Toc185093613)

[Figure 16 Forest plot of Fatigue including Hogg et al.; pre-printed 15](#_Toc185093614)

[Figure 17 Forest plot of UTI including Hogg et al.; pre-printed 15](#_Toc185093615)

[Figure 18 Forest plot of Anxiety including Hogg et al.; pre-printed 15](#_Toc185093616)

[Figure 19 Forest plot of Nausea including Hogg et al.; pre-printed 15](#_Toc185093617)

[Figure 20 Forest plot of Constipation including Hogg et al.; pre-printed 16](#_Toc185093618)

[Figure 21 Forest plot of Vomiting including Hogg et al.; pre-printed 16](#_Toc185093619)

[Figure 22 Forest plot of Diarrhea including Hogg et al.; pre-printed 16](#_Toc185093620)

[Figure 23 Forest plot of Headache including Hogg et al.; pre-printed 16](#_Toc185093621)

# Tables

## Table 1 Search strategy

| **Data Base** | **Search Strategy** |
| --- | --- |
| **PubMed** | **1=** ("Glucagon-Like Peptide-1 Receptor Agonists"[Mesh]) AND "Parkinson Disease"[Mesh])  **2=** ((Glucagon-like peptide-1) OR (GLP-1)) AND (parkinson's disease) AND ((Randomized Controlled Trial) OR (RCT))  **3=** 1 AND 2 |
| **Scopus** | (parkinson*) AND ((GLP-1) OR (GLP-1 agonists) OR (Glucagon-Like Peptide-1 Receptor Agonists) OR (Lixisenatide) OR (Taspoglutide) OR (Liraglutide) OR (Dulaglutide) OR (Exenatide) OR (Semaglutide)) |
| **Web of Science** | (TI=(“Glucagon-Like Peptide-1 Receptor Agonist” OR “Glucagon Like Peptide 1 Receptor Agonist” OR “GLP-1 Receptor Agonist” OR “GLP 1 Receptor Agonist” OR “Incretin Mimetic” OR “GLP-1 Analog” OR “GLP 1 Analog” OR “Exenatide” OR “Liraglutide” OR “Dulaglutide” OR “Semaglutide” OR “Lixisenatide” OR “Albiglutide” OR “Efpeglenatide” )) AND TI=(“Parkinson's Disease” OR “Parkinson Disease” OR “Parkinsonism” OR “Parkinsonian” OR “Paralysis Agitans”) |
| **Clinical Trial.gov** | ("GLP-1 Receptor Agonists" OR "Incretins" OR "liraglutide" OR "exenatide") AND ("Parkinson's Disease" OR "Parkinsonism" OR "Neurodegenerative Disorders") |
| **MEDLINE**  **(OVID)** | **1=** ("Glucagon-Like Peptide-1 Receptor Agonist" or "Glucagon Like Peptide 1 Receptor Agonist" or "GLP-1 Receptor Agonist" or "GLP 1 Receptor Agonist" or "Incretin Mimetic" or "GLP-1 Analog" or "GLP 1 Analog" or "Exenatide" or "Liraglutide" or "Dulaglutide" or "Semaglutide" or "Lixisenatide" or "Albiglutide" or "Efpeglenatide").m_titl.  **2=** ("Parkinson's Disease" or "Parkinson Disease" or "Parkinsonism" or "Parkinsonian" or "Paralysis Agitans").m_titl.  **3=** 1 AND 2 |
| **Cochrane central** | (Parkinson OR "Parkinson's") AND (GLP1 OR GLP OR "Glucagon Like Peptide 1 Receptor Agonists" OR "Glucagon Like Peptide 1" OR "Glucagon-Like Peptide-1 Receptor Agonists" OR "Glucagon-Like Peptide 1 Receptor Agonists" OR "Glucagon-Like Peptide1 Receptor Agonists" OR "Glucagon Like Peptide 1" OR "Glucagon Like Peptide1" OR "Glucagon-Like Peptide-1" OR "Glucagon Like Peptide-1" OR Exenatide OR Byetta OR Bydureon OR Liraglutide OR Victoza OR Saxenda OR Dulaglutide OR Trulicity OR Semaglutide OR Ozempic OR Rybelsus OR Wegovy OR Lixisenatide OR Lyxumia OR Tirzepatide OR Mounjaro OR Efpeglenatide OR Albiglutide OR Tanzeum OR Eperzan OR Cagrilintide OR AM833 OR IDegLira OR IGlarLixi OR Xultophy OR Soliqua) |
| **Google scholar** | Parkinson OR "Parkinson's" AND (GLP1 OR GLP OR "Glucagon Like Peptide 1" OR "Glucagon-Like Peptide-1" OR Exenatide OR Byetta OR Bydureon OR Liraglutide OR Victoza OR Saxenda OR Dulaglutide OR Trulicity OR Semaglutide OR Ozempic OR Rybelsus OR Wegovy OR Lixisenatide OR Lyxumia OR Tirzepatide OR Mounjaro OR Efpeglenatide OR Albiglutide OR Tanzeum OR Eperzan OR Cagrilintide OR AM833 OR IDegLira OR IGlarLixi OR Xultophy OR Soliqua) |

## Table 2.1 Authors judgment with justifications of Athauda et al.; 2017

| **Paper ID** | **Domain** | **Question** | **Author judgement** | **Support for judgment** |
| --- | --- | --- | --- | --- |
| **Athauda et al.; 2017** | **Risk of bias arising from the randomization process** | Random Sequence Generation | YES | “We used SealedEnvelope, an independent, commerical, internetbased randomisation service that generated the online randomisation list on the basis of guidance from the trial IT manager (SH) and trial statistician (SSS)” |
|  |  | Allocation concealment | YES | “The trial statistician (SSS) generated and uploaded unique three-digit identifers for every active and placebo drug kit to the randomisation service to allow allocation of masked study drug kits (sufcient for 12 weeks) at randomisation and follow-up visits by assessing clinicians.” |
|  |  | Baseline balance differences | NO | No baseline differences |
|  | **Risk of bias due to deviations from the intended interventions** | Participants awareness | NO | “Patients and investigators were masked to treatment allocation throughout the study.” |
|  |  | Investigators awareness | NO | “Patients and investigators were masked to treatment allocation throughout the study.” |
|  |  | Appropriate analysis | YES | “Individuals who withdrew before 12 weeks could not contribute data to the primary outcome, and so were replaced per protocol. All 60 patients who completed at least the initial 12-week follow-up were included in the primary analysis.” |
|  |  | Potential impact of the failure to analyze participants | NO |  |
|  | **Risk of bias due to missing outcome data** | Outcome data for all participants | YES | “All efficacy analyses were based on a modified intention-to-treat principle” |
|  | **Risk of bias in measurement of the outcome** | Inappropriate outcome measurement method | NO | “The primary outcome was the adjusted difference in the Movement Disorders Society Unified Parkinson’s Disease Rating Scale (MDS-UPDRS) motor subscale (part 3) in the practically defined off-medication state at 60 weeks.” |
|  |  | Differences between interventional groups | NO | The same outcomes measured in the two groups |
|  |  | Outcome assessor awareness | NO | The trial statistician (SSS) generated and uploaded unique three-digit identifers for every active and placebo drug kit to the randomisation service to allow allocation of masked study drug kits (sufficient for 12 weeks) at randomisation and follow-up visits by assessing clinicians. Patients and investigators were masked to treatment allocation throughout the study. |
|  |  | Assessment of the outcome could have been influenced by knowledge. | NO |  |
|  |  | Assessment of the outcome have been likely influenced by knowledge. | NO |  |
|  | **Risk of bias in selection of the reported result** | Results selected from multiple outcome measurements | NO | All the outcomes that could assess a PD patients are found in the paper. |
|  |  | Results selected from multiple analysis | Probably NO | “All study analyses were done according to a predefined statistical analysis plan.” |
|  |  | Trial analyzed in accordance | Probably YES | “All study analyses were done according to a predefined statistical analysis plan.” |

## Table 2.2 Authors judgment with justifications of Aviles-Olmos et al.; 2013

| **Paper ID** | **Domain** | **Question** | **Author judgement** | **Support for judgment** |
| --- | --- | --- | --- | --- |
| **Aviles-Olmos et al.; 2013** | **Risk of bias arising from the randomization process** | Random Sequence Generation | YES | “Block randomisation will be used with random block sizes. Separate randomisation lists will be generated for patients of greater (Hoehn & Yahr stage 2.5) or lesser (Hoehn & Yahr stage 2.0) disease severity to balance this as a possible prognostic factor” (Found in the protocol section 8.3) |
|  |  | Allocation concealment | YES | Randomisation lists will be created prior to trial commencement, and stored by the trial pharmacist. (See protocol Section 8.3) |
|  |  | Baseline balance differences | NO | “Randomization was stratified by Hoehn and Yahr stage (2, low; 2.5, high) to help ensure similar baseline disability between the 2 groups.” |
|  | **Risk of bias due to deviations from the intended interventions** | Participants awareness | YES | In view of the prohibitive costs associated with manufacture of (QP released) placebo versions of the exenatide pens, these were not available for the purposes of this trial, which was necessarily configured to be open-label from the patient’s perspective. |
|  |  | Investigators awareness | YES | In view of the prohibitive costs associated with manufacture of (QP released) placebo versions of the exenatide pens, these were not available for the purposes of this trial, which was necessarily configured to be open-label from the patient’s perspective. |
|  |  | deviations arose due to experimental context | NO | Patients in both groups continued any licensed PD medication after enrollment and throughout the trial according to the judgement of their treating neurologists. No adjustment of PD medications was made at trial visits unless clinically urgent. |
|  |  | Appropriate analysis | NO | “‘per-protocol’ Patients with- drawing from the trial prior to the 3-month visit were replaced, and new recruits were randomly allocated to the 2 groups” |
|  |  | Potential impact of the failure to analyze participants | Probably NO | 1 drop out < 3 months due to L-dopa dose failures |
|  | **Risk of bias due to missing outcome data** | Outcome data for all participants | YES | “Last observation carried forward” was used for participants with missing data.” |
|  | **Risk of bias in measurement of the outcome** | Inappropriate outcome measurement method | NO | “Their PD was compared after overnight withdrawal of conventional PD medication using blinded video assessment of the Movement Disorders Society Unified Parkinson’s Disease Rating Scale (MDS-UPDRS)” |
|  |  | Differences between interventional groups | NO | The same outcomes measured in the two groups |
|  |  | Outcome assessor awareness | NO | “Assessments of PD severity using MDS-UPDRS part 3 were made “off-medication” after an overnight period and were video recorded to allow objective rating of PD disability by observers blinded to randomization outcomes. Each patient video was rated by the same blinded clinician at each time point.” |
|  |  | Assessment of the outcome could have been influenced by knowledge. | NO |  |
|  |  | Assessment of the outcome have been likely influenced by knowledge. | NO |  |
|  | **Risk of bias in selection of the reported result** | Results selected from multiple outcome measurements | Probably YES | “SCOPA Sleep, SCOPA AUT (they shoud be assessed but we can't find their measures)” |
|  |  | Results selected from multiple analysis | NO | Measure the change values as mentioned in protocol |
|  |  | Trial analyzed in accordance | YES | Section 13 in the protocol |

## Table 2.3 Authors judgment with justifications of McGarry et al.; 2024

| **Paper ID** | **Domain** | **Question** | **Author judgement** | **Support for judgment** |
| --- | --- | --- | --- | --- |
| **McGarry et al.; 2024** | **Risk of bias arising from the randomization process** | Random Sequence Generation | YES | “Participants were randomly allocated (1:1:1) to one of two active treatment groups (2·5 mg or 5·0 mg NLY01) or placebo, using a central computer­generated randomisation scheme with permuted block randomisation and varying block sizes.” |
|  |  | Allocation concealment | YES | “The production randomisation schedule was generated by the Rho Unblinded Interactive Web Response System team and was uploaded into Medidata Randomization and Trial Supply Management. Site staﬀ used this software to randomly allocate participants and receive kit numbers to dispense to them.” |
|  |  | Baseline balance differences | NO | Baseline characteristics were similar across treatment groups (table 1). |
|  | **Risk of bias due to deviations from the intended interventions** | Participants awareness | NO | “Placebo was matched to the study drug for physical characteristics and packaging. All participants, investigators, coordinators, study staﬀ, and sponsor personnel were masked to treatment assignments throughout the study.” |
|  |  | Investigators awareness | NO | “Placebo was matched to the study drug for physical characteristics and packaging. All participants, investigators, coordinators, study staﬀ, and sponsor personnel were masked to treatment assignments throughout the study.” |
|  |  | Appropriate analysis | YES | “The primary analysis of the primary endpoint (treatment difference of least squares mean change from baseline to week 36 in the sum of MDS­UPDRS parts II and III for 2·5 and 5·0 mg NLY01 vs placebo) was conducted in a modified intention­to­treat (mITT) population using a mixed model for repeated measures.” |
|  |  | Potential impact of the failure to analyze participants | NO |  |
|  | **Risk of bias due to missing outcome data** | Outcome data for all participants | YES | intention-to-treat |
|  | **Risk of bias in measurement of the outcome** | Inappropriate outcome measurement method | NO | “The primary efficacy endpoint was the change from baseline to week 36 in the sum of MDS­UPDRS scores for parts II and III. The MDS­UPDRS is a well established and widely used assessment to quantify the signs and symptoms of Parkinson’s disease. The MDS­UPDRS has four parts: part I (non­motor aspects of experiences of daily living), part II (motor aspects of experiences of daily living), part III (motor examination), and part IV (motor complications).” |
|  |  | Differences between interventional groups | NO | The same outcomes measured in the two groups |
|  |  | Outcome assessor awareness | NO | “All participants, investigators, coordinators, study staff, and sponsor personnel were masked to treatment assignments throughout the study.” |
|  |  | Assessment of the outcome could have been influenced by knowledge. | NO |  |
|  |  | Assessment of the outcome have been likely influenced by knowledge. | NO |  |
|  | **Risk of bias in selection of the reported result** | Results selected from multiple outcome measurements | NO | The outcomes reported in protocol were reported in paper |
|  |  | Results selected from multiple analysis | NO | Change in Unified Parkinson's Disease Rating Scale in combined score of Parts II and III from baseline to 36 weeks (protocol) |
|  |  | Trial analyzed in accordance | YES | Section 12 in protocol |

## Table 2.4 Authors judgment with justifications of Meissner et al.; 2024

| **Paper ID** | **Domain** | **Question** | **Author judgement** | **Support for judgment** |
| --- | --- | --- | --- | --- |
| **Meissner et al.; 2024** | **Risk of bias arising from the randomization process** | Random Sequence Generation | YES | “Randomization, performed with the use of a Webbased system, was in unstratified blocks of 4 and 6.” |
|  |  | Allocation concealment | YES | “According to their protocol, researchers stated that concealment was done by the EUCLID trial platform in co-opertation with Sanofi. This allowed the investigatior (which was responsible for the allocation) to get the number corresponding to the treatment to be adminstered to the patient without knowing the group that the patient was allocated to.” (See protocol section 6.3) |
|  |  | Baseline balance differences | NO | “The demographic and clinical characteristics of the participants at baseline were similar in the two groups and were typical of early disease.” |
|  | **Risk of bias due to deviations from the intended interventions** | Participants awareness | NO | All the authors vouch for the completeness and accuracy of the data, the fidelity of the trial to the protocol. **THE PROTOCOL:** “The labels will not provide any information on the type of treatment. Only the treatment number will identify the treatment to be administered to a trial participant. The lixisenatide and placebo are blinded and indistinguishable. The injected volume is a fixed dose of 0.2 mL each. During the double blind treatment period, each treatment kit (and the corresponding pens) is labelled with a treatment number, which is displayed, during the randomization and dispensation process, by the validated web-based system (Ennov Clinical® software) used by the EUCLID clinical trial platform” |
|  |  | Investigators awareness | NO | All the authors vouch for the completeness and accuracy of the data, the fidelity of the trial to the protocol. THE PROTOCOL: “The labels will not provide any information on the type of treatment. Only the treatment number will identify the treatment to be administered to a trial participant. The lixisenatide and placebo are blinded and indistinguishable. The injected volume is a fixed dose of 0.2 mL each. During the double blind treatment period, each treatment kit (and the corresponding pens) is labelled with a treatment number, which is displayed, during the randomization and dispensation process, by the validated web-based system (Ennov Clinical® software) used by the EUCLID clinical trial platform” |
|  |  | Appropriate analysis | YES | “Efficacy and safety end points were assessed in the modified intention-to-treat population, excluding participants with missing data.” |
|  |  | Potential impact of the failure to analyze participants | NO |  |
|  | **Risk of bias due to missing outcome data** | Outcome data for all participants | YES | There are data for 149 participants from 156, the missed data isnt significance (4.5% from data was missed) |
|  | **Risk of bias in measurement of the outcome** | Inappropriate outcome measurement method | NO | “The primary end point was the change from base- line in scores on the Movement Disorder Society–Unified Parkinson’s Disease Rating Scale (MDS-UPDRS) part III” |
|  |  | Differences between interventional groups | NO | The same outcomes measured in the two groups |
|  |  | Outcome assessor awareness | NO | Neither the manuscript nor the protocol stated that there were outcome assessors other than the investigators themselves. Thus, being blinded to allocation and patient groups, they were blinded as regarding outcome assessment. |
|  |  | Assessment of the outcome could have been influenced by knowledge. | NO |  |
|  |  | Assessment of the outcome have been likely influenced by knowledge. | NO |  |
|  | **Risk of bias in selection of the reported result** | Results selected from multiple outcome measurements | NO | The outcomes reported in protocol were reported in paper |
|  |  | Results selected from multiple analysis | NO | “A statistical analysis plan (SAP) will be produced by the statistician before the database freeze” |
|  |  | Trial analyzed in accordance | YES | “A statistical analysis plan (SAP) will be produced by the statistician before the database freeze” |

## Table 2.5 Authors judgment with justifications of Hogg et al.; preprinted

| **Paper ID** | **Domain** | **Question** | **Author judgement** | **Support for judgment** |
| --- | --- | --- | --- | --- |
| **Hogg et al.; pre-printed** | **Risk of bias arising from the randomization process** | Random Sequence Generation | Probably YES | it is only stated in the study "Subjects were randomized by a pre-determined randomization list generated by the biostatistics team." Without showing the way the biostatiscs team made the randomization list; however, this paper is preprinted on Lancet. |
|  |  | Allocation concealment | YES | “The randomization schedule was maintained by the investigational pharmacy, who were the only unblinded study” |
|  |  | Baseline balance differences | Probably NO | “Both motor and cognitive disease severity scores were slightly higher at baseline for subjects randomized to placebo, while non-motor scores were slightly higher in the active treatment group. None of these differences reached statistical significance” |
|  | **Risk of bias due to deviations from the intended interventions** | Participants awareness | NO | The randomization schedule was maintained by the investigational pharmacy, who were the only unblinded study members during the treatment period.The liraglutide and matching placebo injection pens were designed to be identical and visually indistinguishable to the investigators, the subjects, or others assisting in treatment administration. |
|  |  | Investigators awareness | NO | All clinical assessments were only performed by investigators blinded to the treatment allocation of each patient. |
|  |  | Appropriate analysis | YES | Efficacy analyses were based on an intention-to-treat and per- protocol design for patients completing post-randomization follow-up assessments. |
|  |  | Potential impact of the failure to analyze participants | NO |  |
|  | **Risk of bias due to missing outcome data** | Outcome data for all participants | NO | There are data for 51 participants from 63, the missed data is significance (19% from data was missed) |
|  |  | Resulted evidence are not biased | YES | Missing values were imputed using the last observation carried forward (LOCF) method, including clinical scores at discontinuation visit. Baseline values were not carried forward. Analyses of primary and secondary efficacy endpoints were conducted both on the ‘full analysis set’ (FAS) and ‘per protocol’ (PP) population. |
|  | **Risk of bias in measurement of the outcome** | Inappropriate outcome measurement method | NO | Primary outcomes included adjusted difference in the OFF-state Movement Disorders Society Unified PD Rating Scale (MDS-UPDRS) part III, non-motor symptom scale (NMSS) and Mattis Dementia Rating Scale (MDRS-2) at week 54. |
|  |  | Differences between interventional groups | NO | The same outcomes measured in the two groups |
|  |  | Outcome assessor awareness | NO | Blinded clinical assessments were performed by the study investigators at pre-determined intervals |
|  |  | Assessment of the outcome could have been influenced by knowledge. | NO |  |
|  |  | Assessment of the outcome have been likely influenced by knowledge. | NO |  |
|  | **Risk of bias in selection of the reported result** | Results selected from multiple outcome measurements | NO | I can't find its protocol; however, all the outcomes that could assess a PD patients are found in the paper |
|  |  | Results selected from multiple analysis | Probably NO | All study analyses were performed according to a predefined statistical analysis protocol |
|  |  | Trial analyzed in accordance | Probably YES | All study analyses were performed according to a predefined statistical analysis protocol |

# Sensitivity analysis forest plots


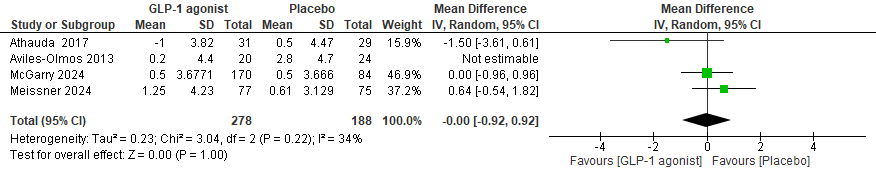


## Figure 1 Sensitivity analysis of MDS-UPDRS I


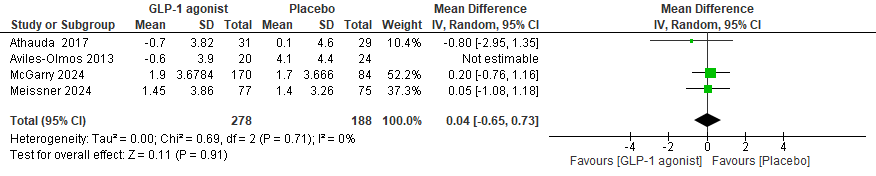


## Figure 2 Sensitivity analysis of MDS-UPDRS II


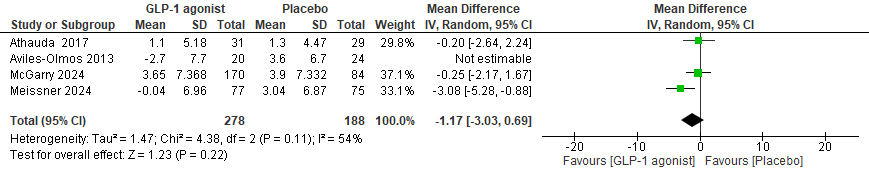


## Figure 3 Sensitivity analysis of MDS-UPDRS III on-medication


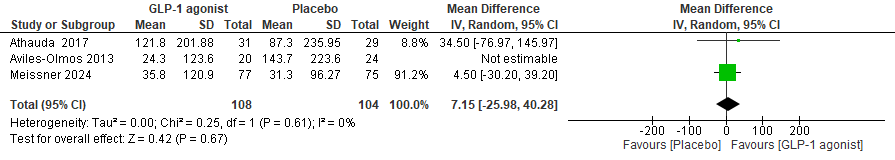


## Figure 4 Sensitivity analysis of LED


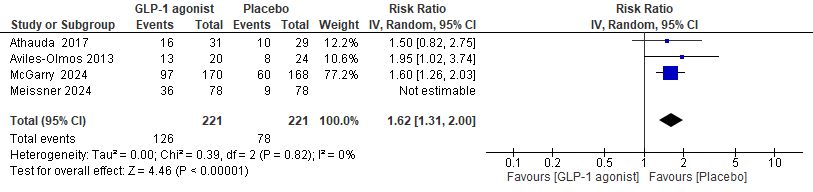


## Figure 5 Sensitivity analysis of Nausea


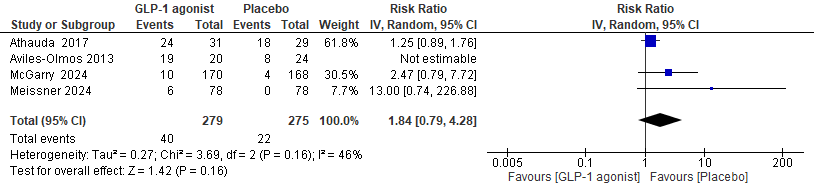


## Figure 6.1 Sensitivity analysis of weight loss (excluding Aviles-Olmos et al.; 2013)


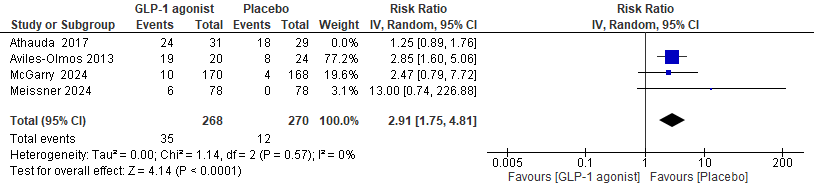


## Figure 5.2 Sensitivity analysis of weight loss (excluding Athauda et al.; 2017)


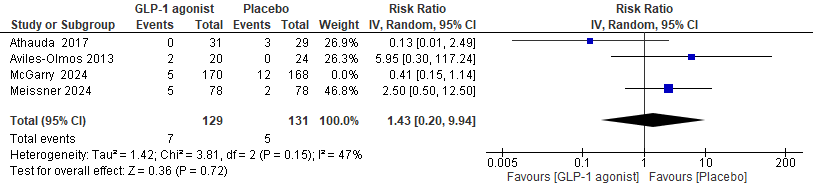


## Figure 7.1 Sensitivity analysis of UTI (excluding McGarry et al.; 2024)


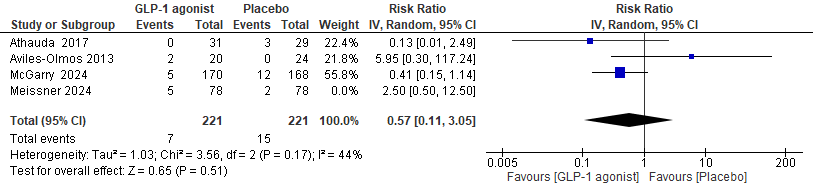


## Figure 6.2 Sensitivity analysis of UTI (excluding Meissner et al.; 2024)

# Meta-analysis including the pre-printed paper


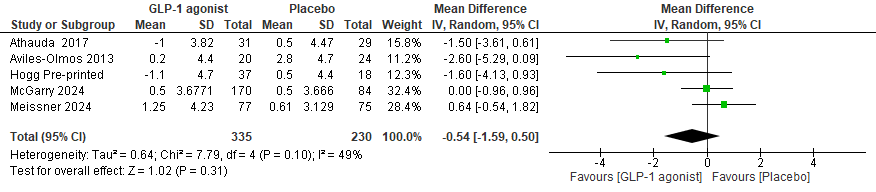


## Figure 8 Forest plot of MDS-UPDRS I including Hogg et al.; pre-printed


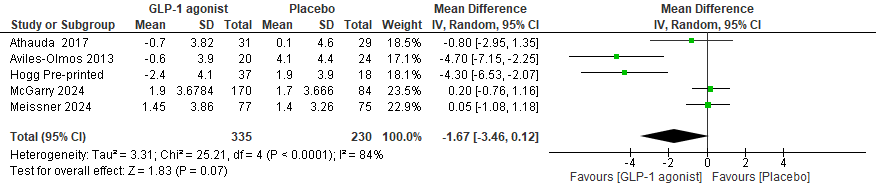


## Figure 9 Forest plot of MDS-UPRDS II including Hogg et al.; pre-printed


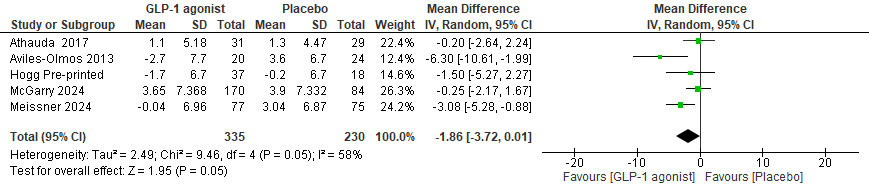


## Figure 10 Forest plot of MDS-UPDRS III On-medication including Hogg et al.; pre-printed


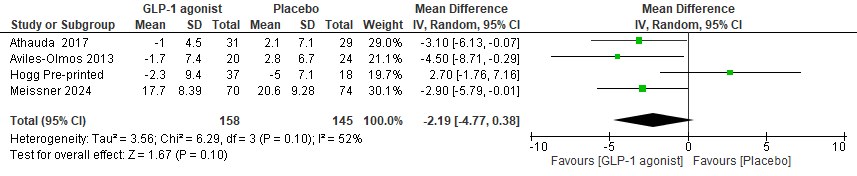


## Figure 11 Forest plot of MDS-UPRDS III Off-medication including Hogg et al.; pre-printed


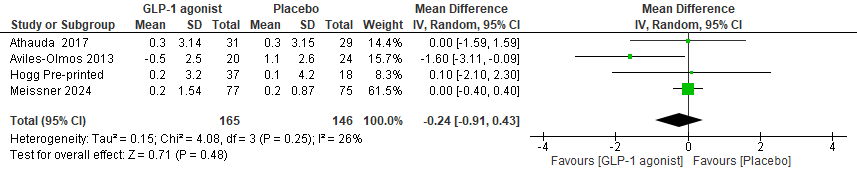


## Figure 12 Forest plot of MDS-UPRDS IV including Hogg et al.; pre-printed


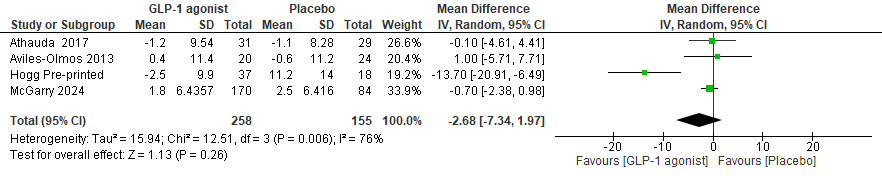


## Figure 13 Forest plot of PDQ-39 including Hogg et al.; pre-printed


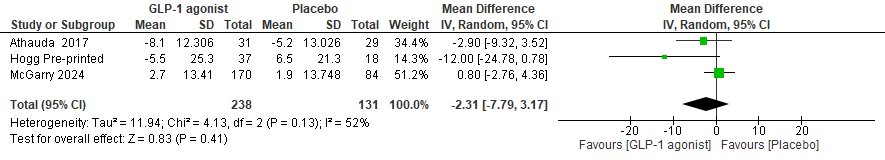


## Figure 14 Forest plot of NMSS including Hogg et al.; pre-printed


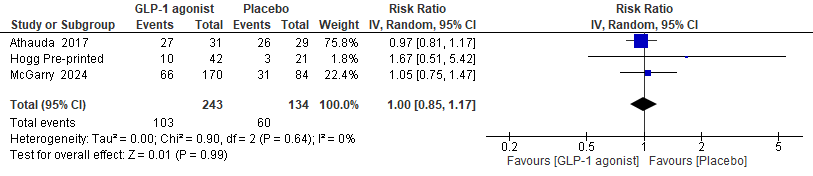


## Figure 15 Forest plot of Administration site disorder including Hogg et al.; pre-printed


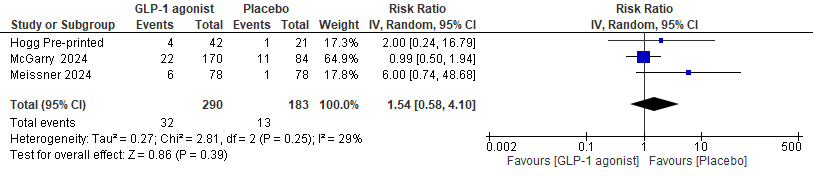


## Figure 16 Forest plot of Fatigue including Hogg et al.; pre-printed


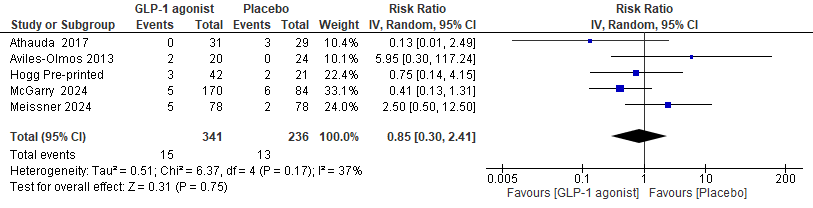


## Figure 17 Forest plot of UTI including Hogg et al.; pre-printed


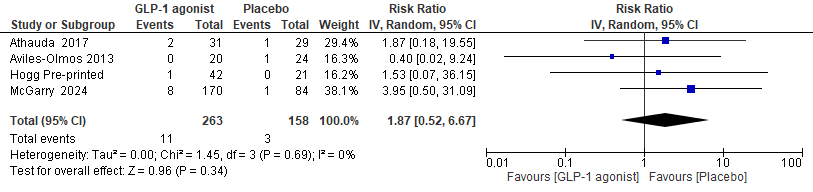


## Figure 18 Forest plot of Anxiety including Hogg et al.; pre-printed


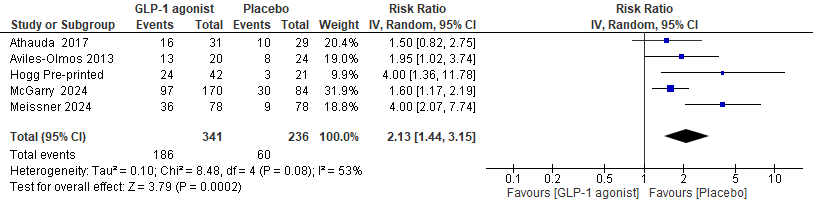


## Figure 19 Forest plot of Nausea including Hogg et al.; pre-printed


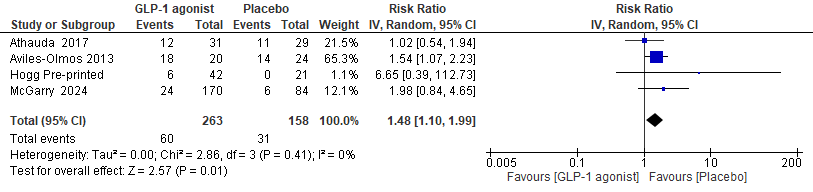


## Figure 20 Forest plot of Constipation including Hogg et al.; pre-printed


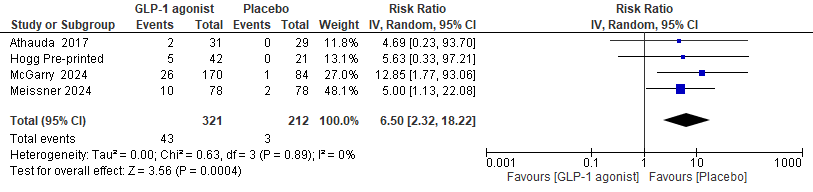


## Figure 21 Forest plot of Vomiting including Hogg et al.; pre-printed


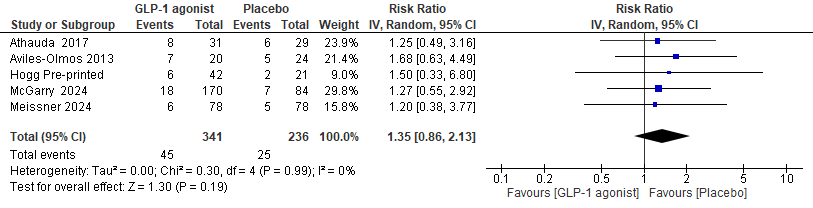


## Figure 22 Forest plot of Diarrhea including Hogg et al.; pre-printed


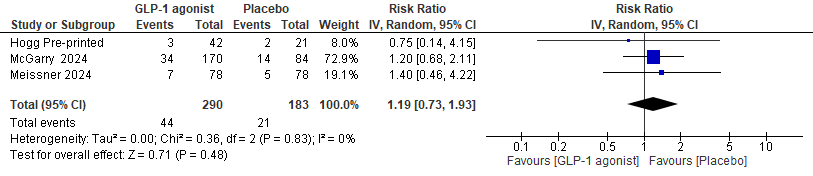


## Figure 23 Forest plot of Headache including Hogg et al.; pre-printed
